# An In-Depth Comparison of Latency-Reversing Agent Combinations in Various *In Vitro* and *Ex Vivo* HIV-1 Latency Models Identified Bryostatin-1+JQ1 and Ingenol-B+JQ1 to Potently Reactivate Viral Gene Expression

**DOI:** 10.1371/journal.ppat.1005063

**Published:** 2015-07-30

**Authors:** Gilles Darcis, Anna Kula, Sophie Bouchat, Koh Fujinaga, Francis Corazza, Amina Ait-Ammar, Nadège Delacourt, Adeline Melard, Kabamba Kabeya, Caroline Vanhulle, Benoit Van Driessche, Jean-Stéphane Gatot, Thomas Cherrier, Luiz F. Pianowski, Lucio Gama, Christian Schwartz, Jorge Vila, Arsène Burny, Nathan Clumeck, Michel Moutschen, Stéphane De Wit, B. Matija Peterlin, Christine Rouzioux, Olivier Rohr, Carine Van Lint

**Affiliations:** 1 Service of Molecular Virology, Institut de Biologie et de Médecine Moléculaires (IBMM), Université Libre de Bruxelles (ULB), Gosselies, Belgium; 2 Service des Maladies Infectieuses, Université de Liège, Centre Hospitalier Universitaire (CHU) de Liège, Domaine Universitaire du Sart-Tilman, Liège, Belgium; 3 Departments of Medicine, Microbiology, and Immunology, University of California, San Francisco, San Francisco, California, United States of America; 4 Laboratory of Immunology, Brugmann University Hospital, Université Libre de Bruxelles (ULB), Bruxelles, Belgium; 5 Institut Universitaire de Technologie Louis Pasteur de Schiltigheim, University of Strasbourg, Schiltigheim, France; 6 Service de Virologie, Université Paris-Descartes, AP-HP, Hôpital Necker-Enfants Malades, Paris, France; 7 Service des Maladies Infectieuses, CHU St-Pierre, ULB, Bruxelles, Belgium; 8 Service de Génétique, Centre Hospitalier Universitaire (CHU) de Liège, Domaine Universitaire du Sart-Tilman, Liège, Belgium; 9 IGBMC (Institut de Génétique et de Biologie Moléculaire et Cellulaire), Illkirch-Graffenstaden, France; 10 Kyolab, Rua Isaura Ap. Oliviera Barbosa Terini, Sao Paulo, Brazil; 11 Department of Molecular and Comparative Pathobiology, Johns Hopkins University School of Medicine, Baltimore, Maryland, United States of America; 12 Institut de Parasitologie et de Pathologie Tropicale, EA7292, University of Strasbourg, University of Strasbourg, Strasbourg, France; Case Western Reserve University School of Medicine, UNITED STATES

## Abstract

The persistence of latently infected cells in patients under combinatory antiretroviral therapy (cART) is a major hurdle to HIV-1 eradication. Strategies to purge these reservoirs are needed and activation of viral gene expression in latently infected cells is one promising strategy. Bromodomain and Extraterminal (BET) bromodomain inhibitors (BETi) are compounds able to reactivate latent proviruses in a positive transcription elongation factor b (P-TEFb)-dependent manner. In this study, we tested the reactivation potential of protein kinase C (PKC) agonists (prostratin, bryostatin-1 and ingenol-B), which are known to activate NF-κB signaling pathway as well as P-TEFb, used alone or in combination with P-TEFb-releasing agents (HMBA and BETi (JQ1, I-BET, I-BET151)). Using *in vitro* HIV-1 post-integration latency model cell lines of T-lymphoid and myeloid lineages, we demonstrated that PKC agonists and P-TEFb-releasing agents alone acted as potent latency-reversing agents (LRAs) and that their combinations led to synergistic activation of HIV-1 expression at the viral mRNA and protein levels. Mechanistically, combined treatments led to higher activations of P-TEFb and NF-κB than the corresponding individual drug treatments. Importantly, we observed in *ex vivo* cultures of CD8+-depleted PBMCs from 35 cART-treated HIV-1+ aviremic patients that the percentage of reactivated cultures following combinatory bryostatin-1+JQ1 treatment was identical to the percentage observed with anti-CD3+anti-CD28 antibodies positive control stimulation. Remarkably, in *ex vivo* cultures of resting CD4+ T cells isolated from 15 HIV-1+ cART-treated aviremic patients, the combinations bryostatin-1+JQ1 and ingenol-B+JQ1 released infectious viruses to levels similar to that obtained with the positive control stimulation. The potent effects of these two combination treatments were already detected 24 hours post-stimulation. These results constitute the first demonstration of LRA combinations exhibiting such a potent effect and represent a proof-of-concept for the co-administration of two different types of LRAs as a potential strategy to reduce the size of the latent HIV-1 reservoirs.

## Author Summary

Persistence of latently infected cells during cART is a major hurdle for HIV-1 eradication. A widely proposed strategy to purge these reservoirs involves the reactivation of latent proviruses. The low levels of active P-TEFb and the cytoplasmic sequestration of NF-κB in resting infected cells largely contribute to maintenance of HIV-1 latency. Therefore, utilization of chemical compounds that target both pathways may lead to more potent effects on HIV-1 reactivation than the effect mediated by the individual drug treatments. In this study, we showed that combined treatments of PKC agonists (prostratin, bryostatin-1 and ing-B) with compounds releasing P-TEFb (JQ1, I-BET, I-BET151 and HMBA) exhibited a synergistic increase in viral reactivation from latency. In-depth comparison of combined treatments in various *in vitro* cellular models of HIV-1 latency as well as in *ex vivo* primary cell cultures from cART-treated HIV^+^ aviremic patients identified bryostatin-1+JQ1 and ing-B+JQ1 to potently reactivate latent HIV-1. The potent effects of these two combinations were detected as early as 24 hours post-treatment. Importantly, bryostatin-1 was used at concentrations below the drug plasma levels achieved by doses used in children with refractory solid tumors. Our mechanistic data established a correlation between potentiated P-TEFb activation and potentiated or synergistic (depending on the HIV-1 latency cellular model used) induction of HIV-1 gene expression observed after the combined versus individual drug treatments. In conclusion, our results establish a proof-of-concept for PKC agonists combined with compounds releasing active P-TEFb as a strategy proposed for a cure or a durable remission of HIV infection.

## Introduction

Recent advances in cART have greatly improved the quality of life for people with HIV-1 infection. However, cART is not curative and patients must stay on therapy indefinitely. Moreover, cART is costly and requires ongoing medical care. Chronic HIV infection, even when suppressed by cART, presents long-term health risks including cancers, cardiovascular diseases or neurocognitive disorders [1,2]. Consequently, achieving either a sterilizing cure (elimination of HIV-1 from the human body) or a remission (a long-term control of HIV in the absence of cART) remains crucial. Persistence of latently infected cells during cART is a major hurdle for HIV-1 eradication [3]. These latently infected cells contain stably integrated, transcriptionally silent but replication-competent proviruses, thereby representing the state of post-integration latency and some of the HIV-1 latent reservoirs. Although many cells may contribute to the latent reservoirs, including monocytes and monocyte-derived macrophages [4,5] (reviewed in [6,7]), the best characterized one is a small population of long-lived HIV-1-infected resting memory CD4^+^ T cells, maintained throughout patient life by homeostatic proliferation and clonal expansion due to specific HIV integration sites [8,9]. The absence of viral gene expression in latently infected cells enables viral escape from host immune system. However, the reservoirs can be induced by various cellular stimuli and therefore represent one potential source of rebound of viremia after cART interruption [10].

Although the discovery of latent reservoirs diminished hopes for eradication, the French VISCONTI study describes patients characterized not only by an extremely low viral reservoir but also by a skewed distribution of this reservoir, whose viremia remains controlled for several years after treatment interruption [11]. Consequently, a decline of the HIV-1 latent reservoir to a level sufficient to permit an efficient control of the infection by the host immune system would represent an important step in order to achieve long-term virological suppression. A widely proposed strategy to reach this goal involves the reactivation of latent proviruses while maintaining cART in order to prevent new spreading infection. This kind of strategy would allow latently infected cells to die from viral cytopathic effect or host immune response. Multiple mechanisms are involved in establishment and maintenance of HIV-1 latency, including (i) epigenetic modifications in the HIV-1 promoter region, (ii) the absence of inducible cellular transcription factors such as NF-κB, (iii) the absence of the HIV-1 transcriptional transactivator (Tat) and Tat-associated factors and (iv) the sequestration of P-TEFb within the 7SK small nuclear ribonucleoprotein (snRNP) repressive complex including the 7SK snRNA, the hexamethylene bisacetamide inducible protein 1 (HEXIM1), the 5’methylphosphate-capping enzyme (MePCE) and the La-related protein (LARP7). The increasing understanding of these mechanisms allowed the identification of LRAs that can abort the state of proviral quiescence and elicit viral expression.

The protein kinase C (PKC) pathway leading to the activation of NF-κB and AP-1 is one of the most important pathway in HIV-1 reactivation (reviewed in [12–14]). Many PKC agonists were considered for purging the reservoirs of latent HIV-1. The phorbol ester prostratin stimulates HIV-1 expression in latently infected T-lymphoid and myeloid cell lines but also in primary cells [15–19] with minimal effects on the immune system [20]. However, the suitability of prostratin for use in humans is still unknown. Bryostatin-1 at low nanomolar concentrations robustly reactivates latent HIV-1 in lymphocytic and monocytic cellular models of post-integration latency and synergizes with histone deacetylase inhibitors (HDACi) to reactivate virus expression in *in vitro* lymphocytic HIV-1 latency models [21,22]. Of note, bryostatin-1 is a PKC agonist that has been tested in more than 20 clinical trials for cancers and Alzheimer’s disease (reviewed in [23]) and showed minimal toxicity [24]. Recently, derivatives of ingenol ester have been considered for reactivation of HIV-1 expression. In particular, ingenol-B (ingenol-3-hexanoate) referred as to ing-B hereafter, was shown to reactivate HIV-1 in *in vitro* latently infected T cell lines and in *ex vivo* CD4^+^ T cells cultures isolated from cART-treated HIV^+^ aviremic patients [25–27]. Concerning safety, ing-B has been evaluated *in vivo* in rats and dogs (Aurigon Life Science, Germany, oral communication) and Rhesus macaques (L. Gama, oral communication) by oral dosing and presented low toxic profile. Importantly, these three PKC agonists exhibit anti-viral activity by downregulating the expression of the HIV-1 receptor CD4 and the coreceptors CXCR4 and CCR5 on the host cell surface, which would lead to the blockade of *de novo* infection [16,22,25].

Although the recruitment of the transcription initiation complex to promoters has long been recognized to play a key role in the regulation of gene expression, the emphasis is now equally placed at subsequent steps of transcription, including elongation. Indeed, the low levels of active P-TEFb in resting CD4^+^ T cells may constitute another major barrier to efficient HIV transcription. Activation of several signaling pathways results in post-translational modifications of P-TEFb subunits and HEXIM-1 [28] which can prevent the sequestration of P-TEFb by the 7SK snRNP complex. Thus, upon cellular activation and when Tat is not produced yet, P-TEFb is released from the 7SK snRNP complex, and associates with the bromodomain-containing protein 4 (BRD4), thereby forming the active P-TEFb complex. P-TEFb is then recruited to the HIV LTR *via* interactions of the BRD4 bromodomains with acetylated histones. Once Tat has been synthesized, on one hand, Tat competes with BRD4 for binding to P-TEFb. On the other hand, Tat is also able to disrupt the inactive P-TEFb complex by displacing HEXIM1 and forming a stable complex with P-TEFb. Tat then recruits P-TEFb to the HIV-1 promoter through TAR and increases transcription elongation [14,29]. Tat can also recruit, in addition to P-TEFb, other elongation factors (such as ELL2, AFF4, ENL and AF9), thereby forming the superelongation complex [30]. Thus, the HIV-1 promoter is critically dependent on levels and activity of P-TEFb. Therefore, the sequestration of P-TEFb in the inactive 7SK snRNP complex is a key factor contributing to HIV latency.

Chemical compounds named Bromodomain and Extraterminal (BET) bromodomain inhibitors (BETi) block the BET bromodomain interaction with acetylated lysine residues. BET-containing protein BRD4 has been shown to be the most specific target of BETi. BETi inhibit BRD4 interaction with P-TEFb and favor the recruitment of PTEFb by Tat to the HIV-1 promoter [31–33]. Moreover, BETi also act by releasing P-TEFb from the 7SK snRNP complex [31]. BETi such as JQ1, I-BET, I-BET151 and MS417 have recently been identified as able to reactivate HIV-1 from latency *in vitro* in several cellular models of HIV-1 post-integration latency, but also *ex vivo* in primary cells isolated from cART-treated HIV^+^ aviremic patients [31,32,34–36]. HMBA (Hexamethylene bisacetamide) is another compound that transiently activates the PI3K/Akt pathway leading to the phosphorylation of HEXIM1 and the subsequent release of P-TEFb from its transcriptionally inactive complex with HEXIM1 and 7SK snRNA [37]. Importantly, PKC agonists have recently been described as involved in P-TEFb regulation not only because they can increase the synthesis of CycT1 and CDK9 in resting CD4^+^ T cells [27,38,39], but also because they can release P-TEFb from the 7SK snRNP complex [40].

In the present study, we demonstrated that individual treatments with P-TEFb releasing compounds (JQ1, I-BET, I-BET151 and HMBA) or PKC agonists (prostratin and bryostatin-1) induced HIV-1 expression which was synergistically increased after combined treatments in several *in vitro* post-integration latency cellular models of T-lymphoid and myeloid lineages. We next compared the reactivation potentials of PKC agonist (including ing-B)+BETi/HMBA co-treatments in *ex vivo* cultures of CD8^+^-depleted PBMCs and of resting CD4^+^ T cells isolated from an high number of cART-treated HIV^+^ aviremic patients. We identified for the first time that co-treatments of JQ1 combined with either bryostatin-1 or ing-B induced extracellular genomic HIV-1 RNA levels to a degree comparable to that obtained after anti-CD3+anti-CD28 antibodies stimulation. The measurements of extracellular genomic HIV-1 RNA levels at multiple time points showed that this potent effect of combined treatments was already detectable one day after stimulation. Therefore, these combinations represent promising candidates for the shock and kill strategy proposed for HIV cure.

Mechanistically, using four independent assays (immunoprecipitations, biomolecular fluorescence complementation assays, reporter gene assays and experiments using various signaling pathways inhibitors), we demonstrated that PKC agonists (such as bryostatin-1) and BETi (such as JQ1) caused a more potent activation of P-TEFb when used in combination than when used individually. Taken together, these mechanistic data established a correlation between the potentiated P-TEFb activation and the potentiated or synergistic (depending on the HIV-1 latency cellular model used) induction of HIV-1 gene expression observed after the combined versus individual LAR treatments. This potentiated release of P-TEFb from the inactive 7SK snRNP complex could explain the potentiated or synergistic activation of HIV-1 gene expression induced by PKC agonist+BETi/HMBA combined treatments. Moreover, the synergistic activation of HIV-1 obtained after cotreatments was dependent on NF-kB.

## Results

### Synergistic activation of HIV-1 production in several *in vitro* latency models by combined treatments of compounds releasing active P-TEFb with either prostratin or bryostatin-1

**Fig 1:**
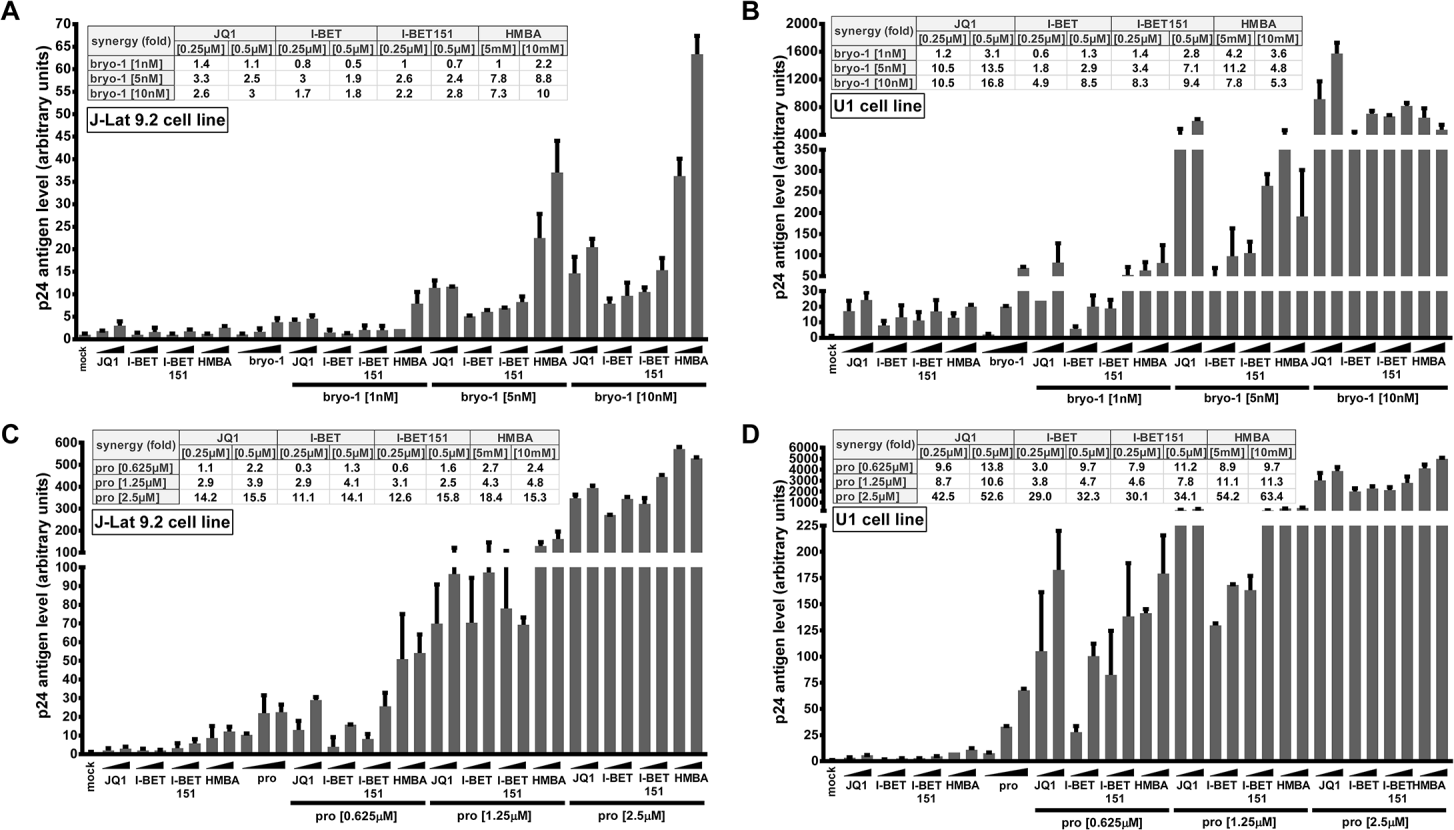
Compounds releasing active P-TEFb and PKC agonists act synergistically to increase HIV-1 production. J-Lat 9.2 (panels **A** and **C**) and U1 (panels **B** and **D**) cell lines were mock-treated or treated with two doses of JQ1, I-BET, I-BET151, HMBA alone or in combination with three doses of either bryostatin-1 or prostratin as indicated. At 24 hours post-treatment, viral production was estimated by measuring CA-p24 antigen concentration in culture supernatants. The mock-treated value was arbitrarily set at a value of 1. Means and standard errors of the means from duplicate samples are indicated. One representative experiment from three is represented. For each combinatory treatment, the fold-synergy was calculated by dividing the effect observed after co-treatments by the sum of the effects obtained after the individual treatments.

To assess whether compounds releasing active P-TEFb (JQ1, I-BET, I-BET151 and HMBA) synergize with PKC agonists (prostratin and bryostatin-1) in reactivating HIV from latency, we first determined their optimal concentrations in terms of both their HIV-1 reactivation potential and their cellular toxicity (S1 Fig). We measured induction of HIV-1 p24 capsid protein production by p24-enzyme-linked immunosorbent assay (ELISA) and we estimated the cell viability by tetrazolium salt-based assay which assesses cell metabolic activity in two well-studied HIV-1 latency cellular models, the T-lymphoid J-Lat 9.2 and promonocytic U1 cell lines. Increasing doses of compounds of interest augmented p24 antigen level in the cell supernatants at 24 hours post-treatment in a dose-dependent manner and caused a decrease in metabolic activity of less than 30% (S1 Fig). Based on our reactivation potential and cell viability data we selected two concentrations of BETi/HMBA and three concentrations of PKC agonists for combinations studies (Fig 1). Two compounds synergize when their combination produces higher effect than the sum of effects arising from separate treatments. We observed a synergistic activation of virus production in the J-Lat 9.2 cell line (Fig 1A and 1C). Bryostatin-1 at 5nM and 10nM produced synergistic increases in p24 production with each dose of BETi/HMBA (Fig 1A). Prostratin at 1.25μM and 2.5μM concentrations also synergized with each dose of BETi/HMBA and the fold-synergy was even higher than the one observed for bryostatin-1+BETi/HMBA combinations (compare Fig 1C to 1A). We also observed high synergistic activations of virus production in the promonocytic U1 cell line (Fig 1B and 1D). Interestingly, synergy in U1 cells was even higher than the one observed in J-Lat 9.2 cell line. Importantly, combined treatments of 10nM bryostatin-1 or 2.5μM prostratin with 0.5μM BETi and 5mM HMBA were the most potent among the tested combinations and produced in most cases the highest synergies in both cellular models. Of note, the same concentrations also produced synergistic activation of virus production in another HIV-1 latency model of promyelocytic origin, the OM10.1 cell line (S2 Fig). The OM10.1 cell line constitutes a more physiological model of HIV latency than the U1 monocytic cell line, since the latter contains HIV proviruses harboring mutations in *tat* gene. Therefore, we chose these drug concentrations for the next reactivation studies.

In conclusion, our results demonstrated that PKC agonists such as prostratin and bryostatin-1 synergistically increased HIV-1 production when combined with BETi/HMBA in lymphocytic and myeloid post-integration latency models, representative of the two main cell types infected by HIV-1 in the natural host.

### Co-treatments PKC agonist+BETi/HMBA increase HIV-1 expression in a higher proportion of cells than the drugs alone and synergistically enhance HIV-1 transcription

**Fig 2:**
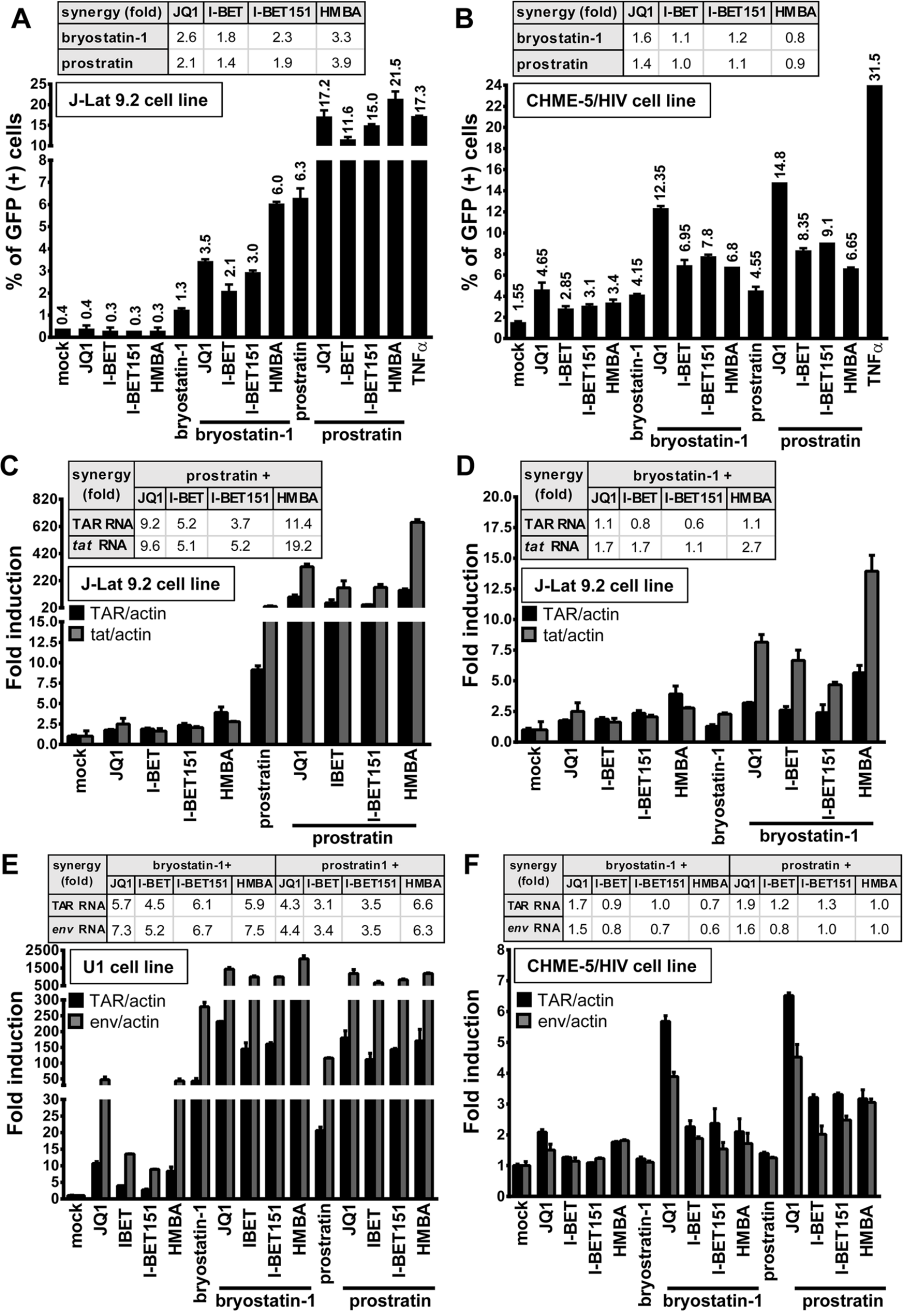
PKC agonist+BETi/HMBA combined treatments increase HIV-1 expression in a higher proportion of cells than the drug alone and synergistically enhance viral transcription. The J-Lat 9.2 cells (panel **A**) or CHME-5/HIV microglial cells (panel **B**) harbor latent HIV-1 provirus containing *gfp* gene. The cells were mock-treated, treated with JQ1 (0.5μM), I-BET (0.5μM), I-BET151 (0.5μM), HMBA (5mM), bryostatin-1 (10nM) and prostratin (2.5μM) alone or in combination as indicated. At 24 hours post-treatment, cells were analyzed by flow cytometry to quantify the proportion of cells expressing GFP. Means and standard errors of the means from duplicate samples are indicated. One representative experiment from three is represented. For each combinatory treatment, the fold-synergy was calculated by dividing the effect observed after co-treatments by the sum of the effects obtained from individual treatments. Panels **C**-**F.** Measurement of initiated and elongated HIV-1 transcripts following drug treatment. Total RNA was extracted from J-Lat 9.2 (panels **C** and **D**), U1 (panel **E**), CHME-5/HIV (panel **F**) cells which were mock-treated or treated with BETi, HMBA, bryostatin-1 and prostratin for 24 hours at concentrations described in Fig 2A and 2B. Initiated (primers TAR) or elongated (primers *tat* or *env*) transcripts were quantified by quantitative real-time RT-PCR. Values were normalized using β-actin gene primers and were presented as fold inductions relative to the values measured in mock-treated cells, which were arbitrarily set at a value of 1. Means and standard errors of the means from duplicate samples are indicated. One representative experiment from two is represented. For each combinatory treatment, the fold-synergy was calculated by dividing the effect observed after co-treatments by the sum of the effects after the individual treatments.

We next investigated whether synergistic effects in viral p24 antigen production (Fig 1) following PKC agonist+BETi/HMBA co-treatments were (i) due to an enhanced HIV-1 expression from those cells whose transcription was already reactivated by the individual drugs or (ii) due to an increase in the number of cells expressing virus. We used the J-Lat 9.2 cell line in which transcriptional activation of the latent provirus can be detected in individual cells by flow cytometry since these cells harbor full-length latent HIV-1 provirus containing *gfp* gene in place of *nef*. In the absence of stimulation, the J-Lat 9.2 cells expressed no GFP, indicating the blockade of viral transcription (Fig 2A). Treatments with each compound releasing P-TEFb used individually did not increase the number of GFP-positive cells (Fig 2A). Bryostatin-1 was weaker than prostratin in inducing GFP expression (1.3% compared to 6.3%). However, when we examined the effects of either bryostatin-1 or prostratin combined with BETi/HMBA, we observed similar synergies. The Jurkat CD4^+^ T-cell-based J-Lat clones are the most studied cellular models of HIV-1 post-integration latency. However, it is critical to address whether similar effects could be observed in other latency models of other cellular origins. For instance, microglial cells are of special importance since they represent the primary host cells for HIV in the brain. Therefore, we took advantage of CHME-5/HIV latently infected microglial cells developed in Jonathan Karn’s laboratory [41]. The CHME-5/HIV cell line containing reporter GFP was treated with prostratin, bryostatin-1, BETi and HMBA alone or in combination. JQ1 was the strongest LRA among the compounds releasing P-TEFb and activated 4.65% of cells (Fig 2B). Conversely, both bryostatin-1 and prostratin activated 4% of cells. Importantly, the proportion of GFP-positive CHME-5/HIV cells was synergistically increased by combined treatments except for the PKC agonist+HMBA and prostratin+I-BET combinations. Combinations of PKC agonist+JQ1 led to the highest synergistic increases in the percentage of GFP-positive cells (12% for bryostatin-1+JQ1 and 15% for prostratin+JQ1).

We next examined the effect of combined treatments in the THP89GFP cell line, a cell line of monocytic origin that is chronically infected with full length HIV-1 expressing GFP [42]. Similarly, we observed that combinations of bryostatin-1+JQ1 and prostratin+JQ1 led to the highest synergistic increases in percentage of GFP-positive cells (82.3% and 94.1%, respectively) (S3A Fig). We also detected synergistic increases in J-Lat cell lines harboring a latent lentiviral construct expressing GFP and expressing or lacking Tat (A2 and A72, respectively) (S3B and S3C Fig, respectively). Thus, our data, in various latency models of T-lymphocytic (J-Lat 9.2, A2 and A72) and myeloid (CHME-5/HIV, THP89GFP) origins, demonstrated that co-treatments of PKC agonist+BETi/HMBA produced an increase in the number of cells expressing virus. We also compared the mean fluorescence intensities (MFI) of the GFP-positive populations following the PKC agonist+BETi/HMBA treatments (S4A–S4C Fig) and we showed that the amount of GFP produced per cell was also synergistically increased. These data showed that synergy was due to both an increase in the number of cells expressing virus and an enhanced HIV-1 gene expression.

Next, we investigated whether the synergistic effects described above were due to enhanced HIV-1 transcription. To this end, we treated J-Lat 9.2 cells with compounds alone or in combination and we quantified initiated and elongated HIV-1 transcripts by quantitative RT-PCR. Combination prostratin+BETi/HMBA produced synergistic increases in relative amounts of both initiated (TAR probe) and elongated (*tat* probe) viral transcripts (Fig 2C). Bryostatin-1 was weaker than prostratin in producing synergistic effects (Fig 2D). In addition, synergistic increases in both initiated (TAR probe) and elongated (*env* probe) viral transcripts were observed in the U1 monocytic cell line (Fig 2E). In CHME-5/HIV microglial cells, synergies were observed for initiated and elongated viral transcripts only for the PKC agonist+JQ1 combinations (Fig 2F), correlating with the data we obtained by flow cytometry (compare Fig 2B and 2F). JQ1 was shown by others to exhibit a good distribution into tissue compartments such as brain and testis which are known HIV-1 sanctuaries [43]. These properties of JQ1 and its ability to reactivate the virus in microglial cells suggest JQ1 as an interesting compound for targeting latent virus in those reservoirs. Importantly, unlike in J-Lat 9.2 cells, in U1 and CHME-5/HIV cells, synergistic effects caused by bryostatin-1 were similar to those caused by prostratin, indicating that combinations can present cell type-dependent specificity and underlying the importance of testing LRAs in various cellular models of HIV-1 latency models of different cellular origins.

Together, our data demonstrated that co-treatments prostratin/bryostatin-1+ BETi/HMBA resulted in diverse synergies that seemed cell-type specific. Moreover, in all cell lines tested, combined treatments PKC agonists+JQ1 were the most potent combinations and increased viral transcripts levels and percentage of activated cells. Combinations of prostratin/bryostratin-1+HMBA led to similar strong synergistic effects, except for treatments in CHME-5/HIV microglial cells.

### The combined treatments have a higher potential than the individual drug treatments in reactivating HIV-1 in CD8^+^-depleted PBMCs and in resting CD4^+^ T cells from cART-treated HIV^+^ aviremic patients

**Fig 3:**
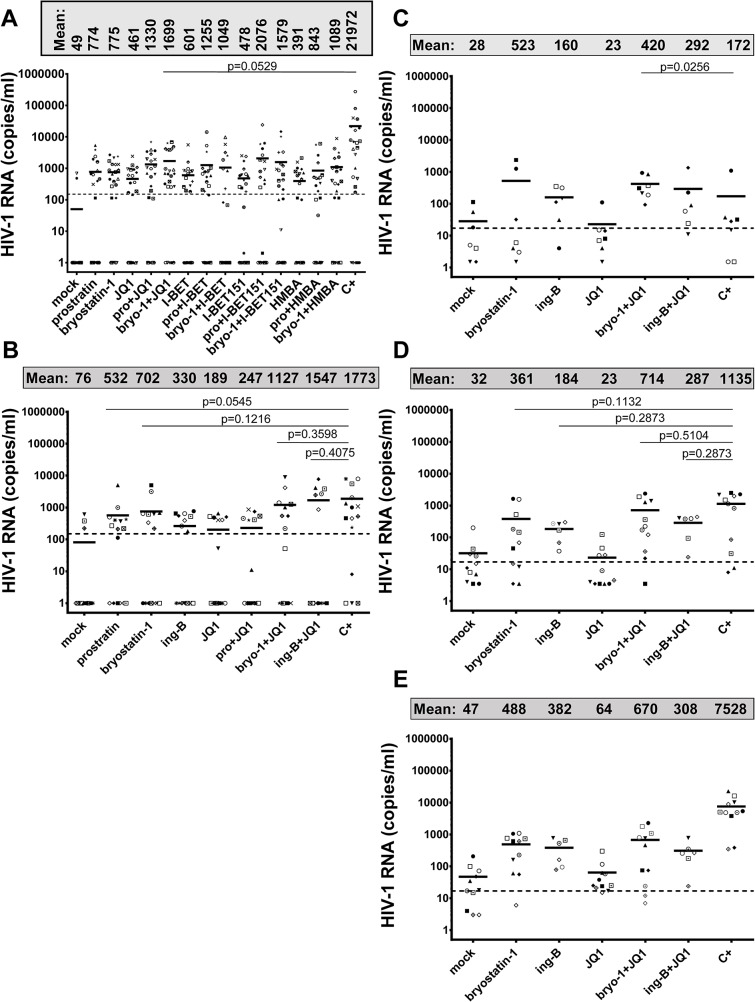
PKC agonists and compounds releasing active P-TEFb induce HIV-1 recovery in CD8^+^-depleted PBMCs and in resting CD4^+^ T cells from cART-treated HIV^+^ aviremic patients. Panel **A.** *Ex vivo* cultures of CD8^+^-depleted PBMCs purified from blood of 24 patients were mock-treated, treated with anti-CD3+anti-CD28 antibodies (C+), prostratin (0.5μM), bryostatin-1 (5nM), JQ1 (0.25μM), I-BET (0.25μM), I-BET151 (0.25μM) or HMBA (5mM) alone or in combination as indicated. Six days post-treatment, concentrations of genomic viral RNA were measured in culture supernatants. Statistical comparisons to positive control are indicated if p<0.05 (superiority to positive control). Panel **B.** *Ex vivo* cultures of resting CD4^+^ T cells purified from blood of 15 patients were mock-treated, treated with anti-CD3+anti-CD28 antibodies (C+), prostratin (0.5μM), bryostatin-1 (5nM), ing-B (10nM), JQ1 (0.25μM), alone or in combination as indicated. Six days post-treatment, concentrations of genomic viral RNA in culture supernatants were determined. Statistical comparisons to positive control are indicated if p>0.05 (non-inferiority). Panels **A** and **B**. Dashed line indicates the 150 HIV-1 RNA copies/ml limit of detection. Panel **C**. *Ex vivo* cultures of CD8^+^-depleted PBMCs purified from blood of 7 HIV^+^ patients were mock-treated, treated with anti-CD3+anti-CD28 antibodies (C+), bryostatin-1 (5nM), JQ1 (0.25μM) or ing-B (10nM) alone or in combination in the presence of cART. One day post-treatment, concentrations of genomic viral RNA in culture supernatants were determined. Statistical comparisons to positive control are indicated if p<0.05 (superiority to positive control). Panel **D.** *Ex vivo* cultures of CD8^+^-depleted PBMCs purified from blood of 11 HIV^+^ patients were mock-treated, treated with anti-CD3+anti-CD28 antibodies (C+), bryostatin-1 (5nM), JQ1 (0.25μM) or ing-B (10nM) alone or in combination in the presence of cART. Three days post-treatment, concentrations of genomic viral RNA in culture supernatants were determined. Statistical comparisons to positive control are indicated if p>0.05 (non-inferiority to positive control). Panel **E.** *Ex vivo* cultures of CD8^+^-depleted PBMCs purified from blood of 11 HIV^+^ patients were mock-treated, treated with anti-CD3+anti-CD28 antibodies (C+), bryostatin-1 (5nM), ing-B (10nM) or JQ1 (0.25μM) alone or in combination in the presence of cART. Six days post-treatment, concentrations of genomic viral RNA in culture supernatants were determined. Statistical comparisons to positive control are not indicated because p<0.05 for all the conditions (superiority of the positive control). Panels **C-E.** Dashed line indicates the 15 HIV-1 RNA copies/ml limit of detection. Panels **A-E.** Each symbol represents one cART-treated HIV^+^ patient. The means are represented.

**Fig 4:**
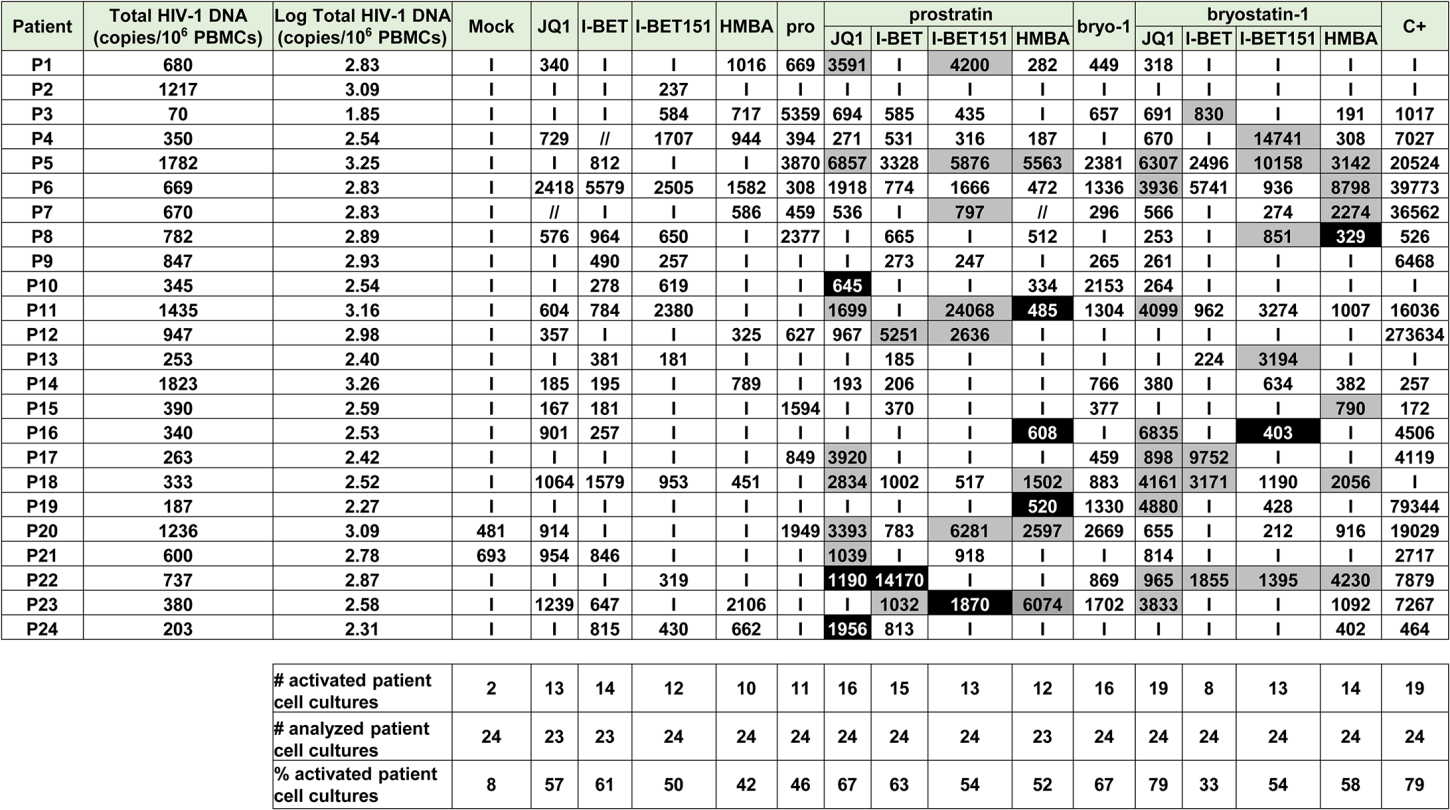
PKC agonists and compounds releasing active P-TEFb induce HIV-1 recovery in CD8^+^-depleted PBMCs from cART-treated HIV^+^ aviremic patients. *Ex vivo* cultures of CD8^+^-depleted PBMCs purified from blood of 24 patients were mock-treated or treated with anti-CD3+anti-CD28 antibodies, prostratin (0.5μM), bryostatin-1 (5nM), JQ1 (0.25μM), I-BET (0.25μM), I-BET151 (0.25μM) or HMBA (5mM) alone or in combination as indicated. Six days post-treatment, concentrations of genomic viral RNA in culture supernatants were determined and the values were expressed as HIV-1 RNA copies/ml. Total HIV-1 DNA was expressed as HIV-1 DNA copies/10^6^ CD8^+^-depleted PBMCs. Values representing higher viral production after the combined treatment than after the single drug treatments are shown in grey. Values representing reactivation of the virus observed exclusively after combined treatments are shown in black. ‘I’ indicates below the 150 HIV-1 RNA copies/ml limit of detection. ‘//’ indicates not tested conditions.

*In vitro* models for HIV-1 latency do not necessarily recapitulate the events occurring during viral latency *ex vivo* [44]. Above we showed high potential in viral reactivation of treatments combining PKC agonists and P-TEFb-releasing agents *in vitro* in HIV-1 latency models of lymphoid and myeloid cell origins. We next determined whether the combined treatments also correlated with HIV-1 recovery in *ex vivo* cultures of CD8^+^-depleted PBMCs and resting CD4^+^ T cells isolated from cART-treated aviremic HIV-1^+^ patients. Firstly, we evaluated cellular viability in CD8^+^-depleted PBMCs cultures purified from blood of 5 uninfected donors following drug treatments (S5 Fig). BETi, HMBA and bryostatin-1 did not alter cellular metabolic activity at any concentration tested. However, we observed a dose-dependent decrease in metabolic activity in prostratin-treated CD8^+^-depleted PBMCs with a metabolic activity of 25% for the above used prostratin concentration (2.5μM). Therefore, we chose for further experiments a dose of prostratin (0.5μM) presenting a cellular metabolic activity of 60%. In order to evaluate HIV-1 recovery, CD8^+^-depleted PBMCs purified from blood of 24 cART-treated HIV^+^ aviremic patients were mock-treated, treated with anti-CD3+anti-CD28 antibodies as a positive control for global T cell activation or with indicated LARs (Fig 3A, Fig 4 and S1 Table). Cell-associated total HIV-1 DNA and extracellular viral RNA were quantified. The number of proviruses seeded in each well was estimated from the number of HIV-1 DNA copies/10^6^ cells determined by qPCR assuming negligible unintegrated HIV-1 DNA in patients after long-term cART [8]. Measurements of extracellular HIV RNA represents a surrogate marker for virions release from treated cell cultures. Indeed, Mellors’ group has highlighted the necessity to measure viral production and not only unspliced cellular HIV-1 RNA following HIV-1 reactivation since these authors did not observe significant correlation between these two methods after treatment with an other LRA, the HDACi, SAHA [45]. We assumed that extracellular virions would accumulate over the course of several days; therefore, the duration of culture was extended to 6 days to maximize assay sensitivity.

We detected HIV-1 RNA in 2 out of 24 patient’s cultures (8%) in mock-treated conditions. This phenomenon could be explained by the stochastic distribution of HIV^+^ cells in the population of isolated cells and by the activation of those HIV^+^ cells during the experiment. I-BET led to reactivation of 61% of the patient cell cultures (Fig 4) and caused an increase in recovered viral genomic RNA (mean of 601 HIV-1 RNA copies/ml). JQ1 led to reactivation of 57% of the patient cell cultures (Fig 4) and caused an increase in recovered viral genomic RNA (mean of 461 HIV-1 RNA copies/ml). I-BET151 and HMBA increased the percentage of reactivated cultures (50 and 42%, respectively) and the mean levels of extracellular HIV-1 RNA were 478 and 391 HIV RNA copies/ml, respectively (Fig 4). Bryostatin-1 was more potent than prostratin and led to reactivation of 67% of cultures when the latter reactivated 46% of cultures. Bryostatin-1 and prostratin produced similar increases in viral RNA levels (mean of 755 HIV-1 RNA copies/ml and of 774 HIV-1 RNA copies/ml, respectively). Combined treatments of prostratin+BETi/HMBA produced increases in the percentage of reactivated cultures as compared to individual drug treatments (Fig 4). Among the bryostatin-1 combined treatments, only bryostatin-1+JQ1 produced an increase in percentage of activated cultures. From the combined treatments tested, prostratin+JQ1 and bryostatin-1+JQ1 presented the highest percentages of reactivated cultures (67 and 79%, respectively). Importantly, HIV-1 recovery obtained after bryostatin-1+JQ1 co-treatment (79%) reached the same percentage increase as the one obtained with the positive control (79%). Notably, all combinations of PKC agonist+compound releasing P-TEFb produced higher mean levels of extracellular HIV-1 RNA than the mean levels obtained with individual drug treatments (Fig 3A). We performed statistical analysis of the reactivation levels observed after individual and combined treatments compared to the level observed after anti-CD3+anti-CD28 stimulation (Fig 3A). Remarkably, the level obtained after the combination bryostatin-1+JQ1 was the only one to exhibit statistical non-inferiority to the positive control level (Fig 3A, p>0.05). This result confirmed the potency we demonstrated in Fig 4 in terms of frequency of reactivated patient cell cultures.

In addition to the increases in the reactivation frequency and in mean extracellular HIV-1 RNA levels, we demonstrated a higher viral production in several cell cultures following combined treatments. For instance, as shown in Fig 4, HIV-1 recovery was only observed with the combination but not with the two drugs used separately (Fig 4, see black boxes). Moreover, in several *ex vivo* cultures, combined treatments allowed higher levels of viral production than the levels observed with the individual treatments (Fig 4, see grey boxes).

**Fig 5:**
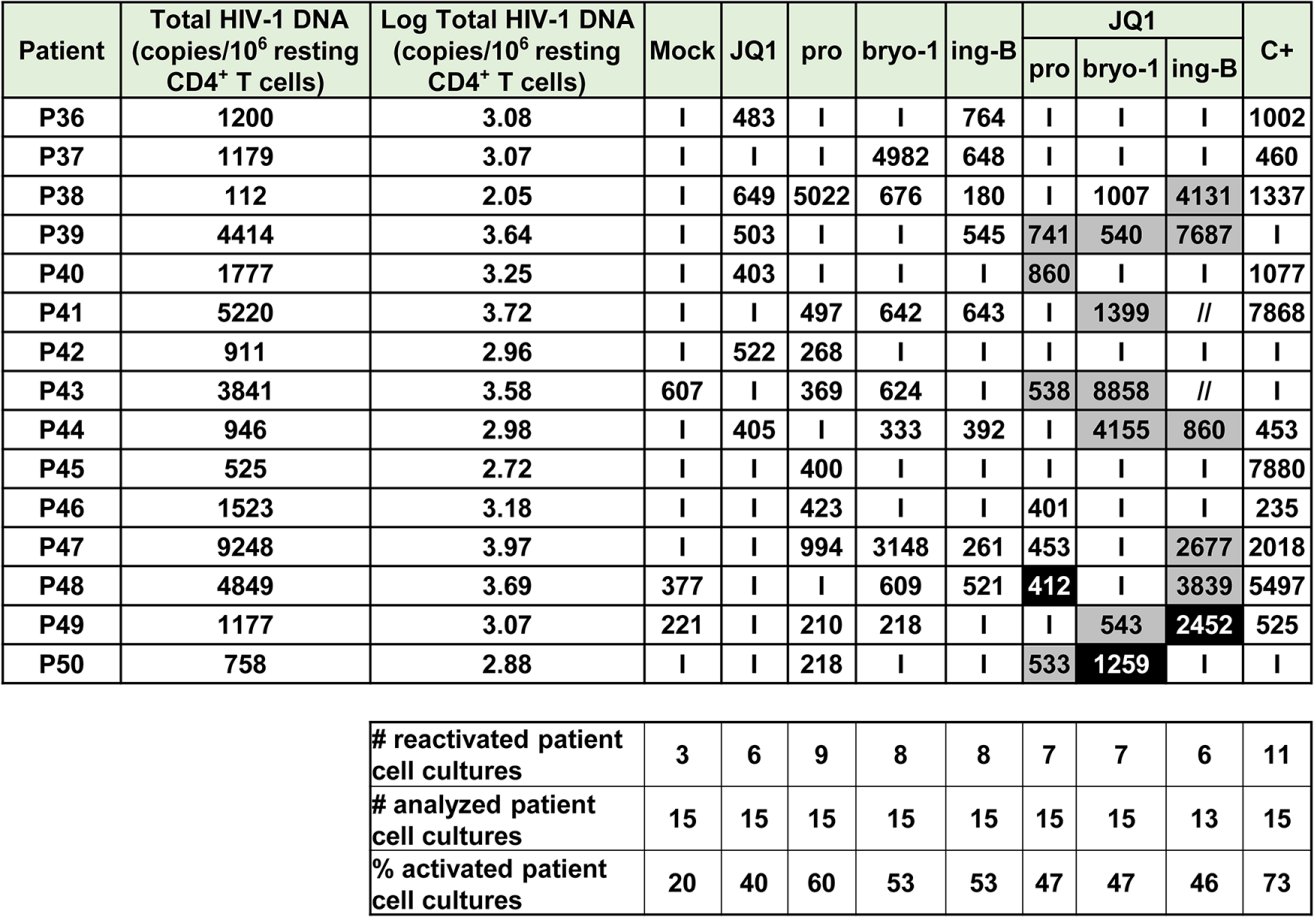
PKC agonists and JQ1 induce HIV-1 recovery in resting CD4^+^ T cells from cART-treated HIV^+^ aviremic patients. *Ex vivo* cultures of resting CD4^+^ T cells purified from blood of 15 patients were mock-treated or treated with anti-CD3+anti-CD28 antibodies, JQ1(0.25μM), prostratin (0.5μM), bryostatin-1 (5nM) or ing-B (10nM) alone or in combination as indicated. Six days post-treatment, concentrations of viral RNA in culture supernatants were determined and the values were expressed as HIV-1 RNA copies/ml. Total HIV-1 DNA was expressed as HIV-1 DNA copies/10^6^ resting CD4^+^ T cells. Values representing higher viral production after a combinatory treatment than after the corresponding single drug treatments are shown in grey. Values representing reactivation of viral production observed exclusively after combined treatment are shown in black. ‘I’ indicates below the 150 HIV-1 RNA copies/ml limit of detection. ‘//’ indicates not tested conditions.

Among the cell types present in CD8^+^-depleted PBMCs, latently infected resting CD4^+^ T cells represent the major reservoir of HIV infection. We therefore evaluated the reactivation potential of individual and combined treatments in this cell type. Fig 3A and Fig 4 showed that PKC agonist+JQ1 co-treatments led to (i) the highest percentages of reactivated patient cell cultures and (ii) important increases in extracellular viral RNA compared to other PKC agonist+BETi/HMBA co-treatments in CD8^+^-depleted PBMCs purified from blood of cART-treated HIV^+^ aviremic patients. Therefore, we evaluated in resting CD4^+^ T cells the reactivation potentials of JQ1 alone or combined with either prostratin or bryostatin-1 or another PKC agonist, ing-B. Indeed, while we were performing this study, ing-B has been reported to potently reactivate latent HIV-1 [25,26] *in vitro* and we decided to evaluate here its potential *ex vivo*. We isolated HLA-DR^-^, CD69^-^, CD25^-^ CD4^+^ T cells from blood of 15 cART-treated HIV^+^ aviremic patients. *Ex vivo* cell cultures were mock-treated, treated with anti-CD3+anti-CD28 antibodies or with the compounds of interest (Fig 5 and S1 Table). Cell-associated total HIV-1 DNA and extracellular viral RNA were quantified. We detected HIV-1 RNA in 3 out of 15 patient’s cultures (20%) in mock-treated conditions. Prostratin, bryostatin-1, ing-B, and JQ1 reactivated latent HIV-1 in 60%, 53%, 53%, and 40% of the cultures, respectively (Fig 5). All individual treatments produced increases in mean HIV-1 RNA copies/ml levels that were higher than the 150 copies/ml threshold (Fig 3B). Notably, the combination bryostatin-1+JQ1 and ing-B+JQ1 produced higher increases in mean viral RNA levels relative to the individual treatments. These bryostatin-1+JQ1 and ing-B+JQ1-induced increases reached 1127 and 1547 HIV-1 RNA copies/ml, respectively (Fig 5). Importantly, the very high levels of reactivated virus following those treatments were statistically non-inferior (p>0.05) to the level obtained after anti-CD3+anti-CD28 stimulation. In contrast, we did not observe any benefit of combining prostratin with JQ1. All the combined treatments did not increase the frequency of reactivated patient cell cultures relative to the individual treatments, but synergistically increased HIV-1 recovery in most of the cultures (Fig 5, see black and grey boxes). For instance, the ing-B+JQ1 co-treatment led to reactivation in 46% of *ex vivo* cultures and, remarkably, all these cultures exhibited strong synergistic increases. Of note, we also performed individual and combined treatments using ing-B in CD8^+^-depleted PBMCs from cART-treated HIV^+^ aviremic patients and we observed strong reactivations of latent HIV-1. This part of our work concerning ing-B individual treatment of patient cell cultures will be reported in a separate study (L. Gama and colleagues, manuscript in preparation).

Consequently, our results established that PKC agonists acted as powerful LRAs and that their reactivation properties were potentiated when used in combination with P-TEFb-releasing agents, especially JQ1. However, it should be noted that we did not observe viral reactivation either after individual, combined or anti-CD3+anti-CD28 treatments in all the patient cell cultures. This observation can be explained by the multifactorial and stochastic nature of viral latency but also by the very low frequency of latently infected cells carrying replication competent proviruses. Indeed, in the experiments described above, we plated 6 million CD8^+^-depleted PBMCs or 0.5 million resting CD4+ T cells per culture condition. Assuming the scarcity of latently-infected cells, it is possible that some of the experimental wells did not contain any reservoir. Moreover, we used a reactivation system without cART in order to increase the sensitivity of our assays since it allowed infection of new cells and multiple viral cycles. Nevertheless, it may have introduced a bias since the compounds used may have sensitized bystander cells to new infections. Based on the results presented in Fig 3A and 3B, we thus selected the most potent drug combinations (bryostatin-1+JQ1 and ing-B+JQ1) and tested them in the presence of cART. We plated 12 million of CD8^+^-depleted PBMCs in order to increase the number of latently infected cells per tested condition. Moreover, we looked at the effect of the drugs not only at day 6 but also at day 1 and day 3 of culture. Of note, continuous bryostatin-1 infusion of 24 hours [46] and 72 hours [47] were previously tested in patients affected by malignancies.

**Fig 6:**
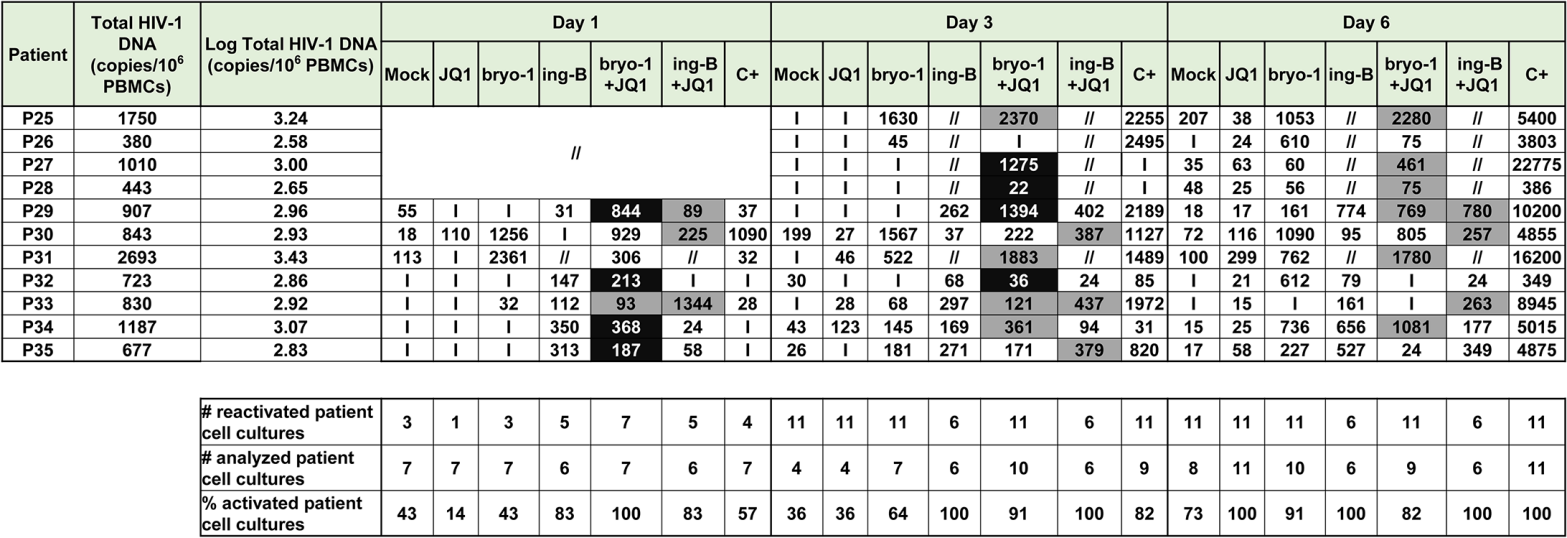
PKC agonists and JQ1 induce HIV-1 recovery in CD8^+^-depleted PBMCs from cART-treated HIV^+^ aviremic patients in the presence of cART. *Ex vivo* cultures of CD8^+^-depleted PBMCs purified from blood of 11 patients were mock-treated or treated with anti-CD3+anti-CD28 antibodies, JQ1 (0.25μM), bryostatin-1 (5nM) or ing-B (10nM) alone or in combination as indicated. Concentrations of viral RNA in culture supernatants were determined one day, three days or six days post-treatment. The values were expressed as HIV-1 RNA copies/ml. Total HIV-1 DNA was expressed as HIV-1 DNA copies/10^6^ CD8^+^-depleted PBMCs. Values representing higher viral production after a combinatory treatment than after the corresponding single drug treatments are shown in grey. Values representing reactivation of virus production observed exclusively after combined treatments are shown in black. ‘I’ indicates below the 15 HIV-1 RNA copies/ml limit of detection, ‘//’ indicates not tested conditions.

At day 1 (Fig 6 and S1 Table), the combination bryostatin-1+JQ1 was the only condition that caused viral reactivation in the cell cultures from 7 patients tested (100% of reactivation). Surprisingly, this combined treatment exceeded the effect observed after anti-CD3+anti-CD28 stimulation (p = 0.0256) (Fig 3C). The combination ing-B+JQ1 reactivated latent HIV-1 in 83% of cultures, a result identical to that observed with ing-B alone and that exceeded the percentage obtained after stimulation with the positive control (57%) (Fig 6). Importantly, this ing-B+JQ1 combination resulted in a higher mean level of extracellular HIV-1 RNA than the mean level obtained with ing-B alone (mean of 292 HIV-1 RNA copies/ml and of 160 HIV-1 RNA copies/ml, respectively (Fig 3C)).

At day 3 (Fig 6 and S1 Table), the combination bryostatin-1+JQ1 reactivated HIV-1 in 10 out of 11 patient cultures (91% of reactivated patient cell cultures). This percentage was thus higher than percentages obtained after treatment with bryostatin-1 alone (64%) or after the positive control stimulation (82%). The conditions ing-B alone and ing-B+JQ1 reactivated HIV-1 in 100% of the *ex vivo* cultures. Similarly to the results obtained at day 1, ing-B+JQ1combination resulted in a higher mean level of extracellular HIV-1 RNA than the mean level obtained with ing-B alone (mean of 287 HIV-1 RNA copies/ml and of 184 HIV-1 RNA copies/ml, respectively) (Fig 3D). As indicated in Fig 3D, the levels observed after the individual PKC agonist treatments and after the combinations PKC agonists+JQ1 were at day 3 statistically non-inferior (p>0.05) to the level obtained after anti-CD3+anti-CD28 stimulation.

At day 6 (Fig 3E, Fig 6 and S1 Table), the sensitivity of the RNA quantification allowed us to detect a viral production at very low level even in the mock-treated cultures, probably due to the activation of HIV^+^ cells during the experiment. It is consequently difficult to discuss the percentages of reactivation observed. The combination bryostatin-1+JQ1 produced a higher mean level of extracellular HIV-1 RNA than the mean level obtained with bryostatin-1 alone (mean of 670 HIV-1 RNA copies/ml and of 382 HIV-1 RNA copies/ml, respectively), confirming in the presence of cART the results presented in Fig 3A in the absence of cART. The combination ing-B+JQ1 produced a slightly lower mean level of extracellular HIV-1 RNA than the mean level obtained with ing-B alone. At day 6, the positive control treatment showed a statistical superiority to all the tested conditions. Indeed, unlike the stimulation by the LRAs, the stimulation by the positive control relied on continuous cell stimulation arising from coated anti-CD3 antibodies. This could explain that the level of extracellular genomic viral RNA was increasing in a time-dependent manner. On the contrary, the maximal effect of the bryostatin-1+JQ1 and ing-B+JQ1 combinations appeared earlier (at day 3 and day 1, respectively). Importantly, the kinetics of the effect observed with bryostatin-1 corresponded to its clinical use [46,47]. Interestingly, JQ1 did not lead to a significant viral reactivation in the presence of cART (Fig 3C–3E), probably because the reactivation potential of this LRA is weak when used alone and needs to be amplified in order to be detected.

Moreover, we evaluated the cell viability for PKC agonist+JQ1 combinations in CD8^+^-depleted PBMCs isolated from blood of uninfected donors and we showed that prostratin+JQ1, bryostatin-1+JQ1 and ing-B+JQ1 combinations presented 82%, 62% and 69% of cellular metabolic activity, respectively (S6A Fig) and caused no cell death (S6B Fig).

Finally, in order to verify that the reactivated viruses were infectious, we performed infections of Jurkat cells with viruses isolated from bryostatin-1+JQ1 treated patient cell cultures and demonstrated the infectivity of the reactivated viruses (S2 Table).

In conclusion, our results established that PKC agonists acted as powerful LRAs and that their reactivation properties were potentiated when used in combination with compounds releasing P-TEFb and thereby increasing transcriptional elongation, especially JQ1. Importantly, HIV-1 reactivation potencies of these combinations were already clearly detected at 24 hours post-treatment.

### Synergistic HIV-1 recovery following PKC agonist+BETi/HMBA combined treatments is dependent on NF-κB

**Fig 7:**
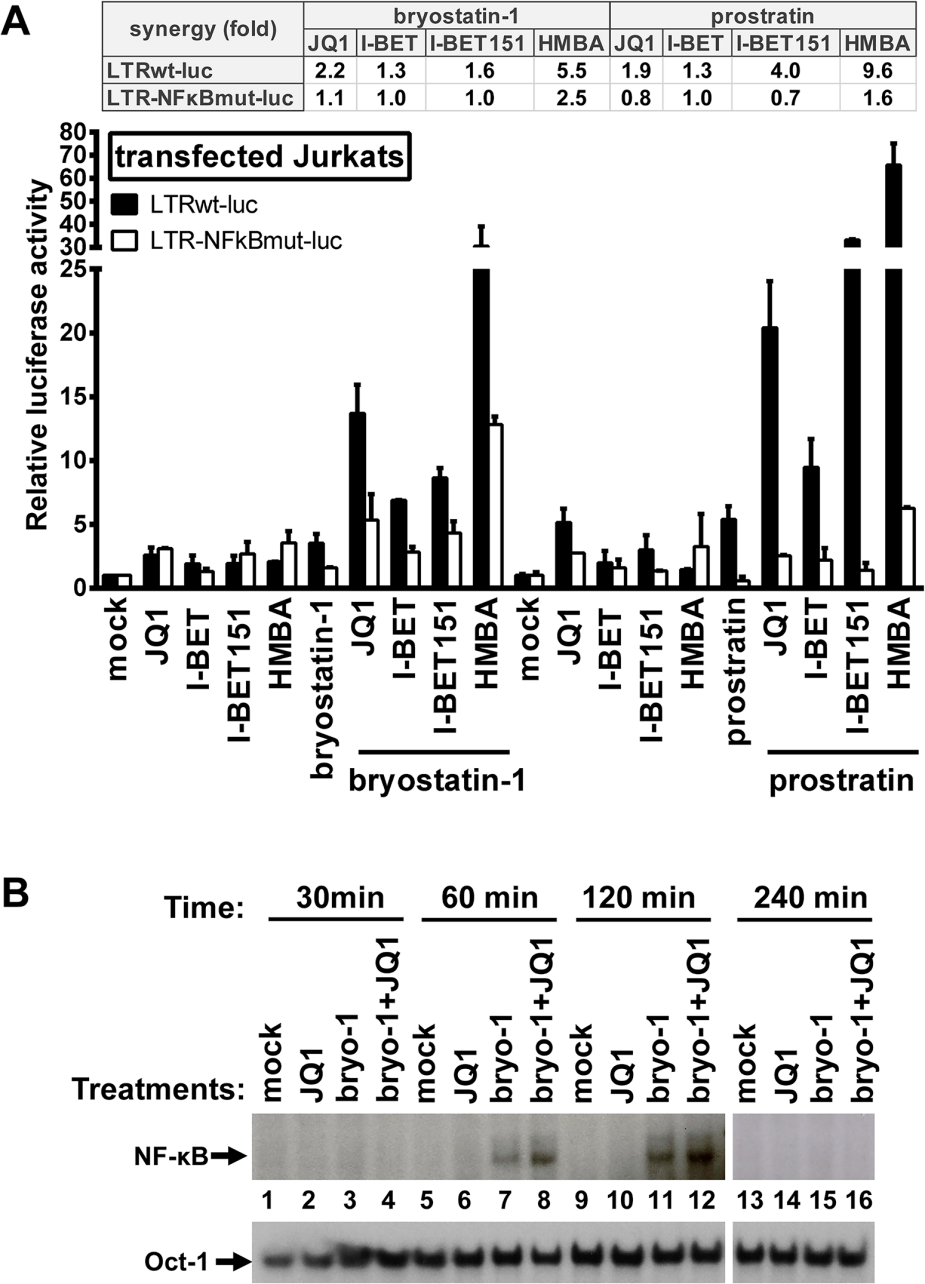
Synergistic inducibility of HIV-1 LTR promoter by PKC agonist+BETi/HMBA combinatory treatments depends on NF-κB. Panel **A.** Jurkat cells were transiently transfected with the episomal plasmid containing the luciferase reporter gene driven either by the wild-type HIV LTR promoter (LTRwt-luc) or by the LTR promoter mutated in the two NF-κB binding sites (LTR-NFκBmut-luc). Twenty-four hours later, cells were mock-treated, treated with JQ1 (0.5μM), I-BET (0.5μM), I-BET151 (0.5μM), HMBA (5mM), bryostatin-1 (10nM) and prostratin (2.5μM) alone or in combination. Luciferase activities in cell extracts were measured 24 hours after drug treatments and reported as fold increases over the activity observed in mock-treated conditions (transfection of the reporter plasmid without drug treatment) and arbitrarily set at values of 1. An experiment performed in triplicates representative of two independent experiments is shown. Panel **B.** Nuclear extracts were prepared from Jurkat cells which were mock-treated, treated with bryostatin-1 (10nM), JQ1 (0.5μM) or with bryostatin-1+JQ1 for different time periods. An oligonucleotide corresponding to the HIV-1 LTR NF-κB sites was used as probe in EMSAs. As control for equal loading, the bottom panel shows comparability of the various nuclear extracts assessed by EMSA with an Oct-1 consensus probe.

We assessed the molecular mechanisms underlying the synergistic induction of HIV-1 observed at the viral RNA and protein levels following individual or combined treatments with PKC agonists and BETi/HMBA (Figs 1–6). Firstly, we evaluated the involvement of two NF-κB binding sites located in the enhancer region of the HIV-1 promoter. We transiently transfected Jurkat cells either with episomal vector driving the luciferase expression from wild-type LTR (LTRwt-luc) or with similar reporter vector mutated in two NF-κB binding sites located in HIV LTR (LTR-NFκBmut-luc). Cells were stimulated with JQ1, I-BET, I-BET151, HMBA, prostratin, and bryostatin-1 alone or in combination. Combinations of PKC agonist+BETi/HMBA led to synergistic increases in luciferase activity arising from LTRwt-luc transfection (Fig 7A). We observed that lack of the two NF-κB binding sites located in HIV-1 LTR impaired the synergistic increase in luciferase activity following combined treatments. These data indicated the involvement of NF-κB protein binding to the viral promoter in the PKC agonist+BETi/HMBA synergies. To assess the effect of the drugs on the NF-κB binding activity, we next performed electrophoretic mobility shift assays (EMSAs) using an HIV-1 NF-κB probe and nuclear extracts from Jurkat T cells stimulated for various periods of time with JQ1, bryostatin-1 or bryostatin-1+JQ1 (Fig 7B). We detected an induction of NF-κB DNA-binding activity in response to a 60 min treatment with bryostatin-1 (Fig 7B, lane 7). JQ1 alone caused no induction of NF-κB binding activity even after a 240 min treatment (Fig 7B, lanes 2, 6, 10 and 14). When bryostatin-1 was combined with JQ1, an induction of NF-κB binding activity stronger than that obtained with bryostatin-1 alone was observed at 60 and 120 min time points (Fig 7B, compare lane 7 with lane 8 and lane 11 with lane 12). This effect faded away at 240 min time point, confirming transient NF-κB DNA- binding activity. Our results demonstrated that JQ1 increased bryostatin-1-induced NF-κB DNA-binding activity. This could explain, at least in part, the synergistic increases in HIV transcription following combined treatments observed in Fig 2.

In conclusion, our data indicated that the synergistic activation of HIV-1 obtained after PKC agonist+BETi/HMBA combinationed cellular treatments was, at least in part, dependent on NF-κB.

### Bryostatin-1+JQ1 combination has a higher potential in activating P-TEFb than the individual drug treatments

**Fig 8:**
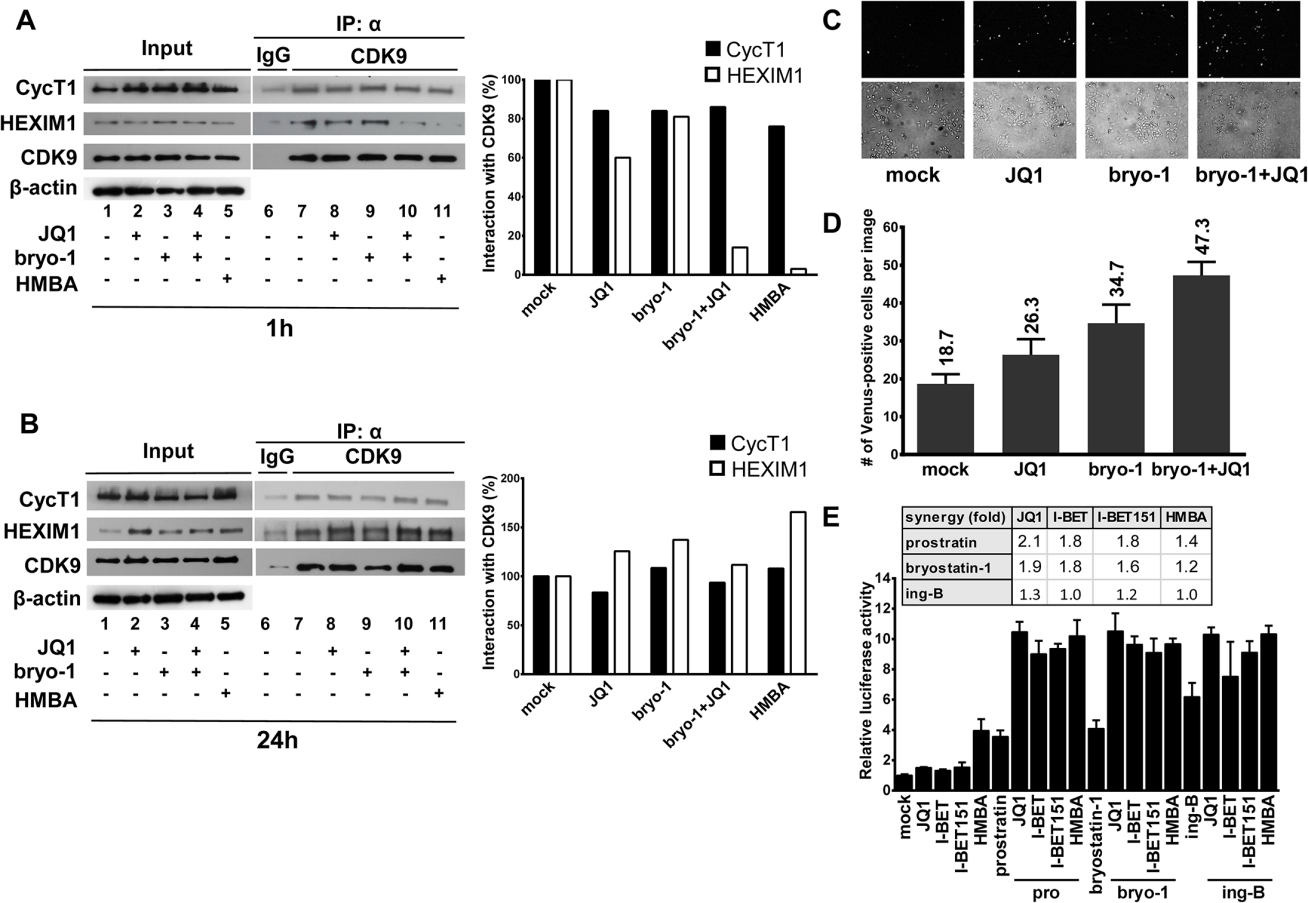
PKC agonists synergize with BETi in releasing active P-TEFb. Jurkat cells were mock-treated, treated with JQ1 (0.25μM), bryostatin-1 (5nM) alone or in combination for 1 hour (Panel **A**) or 24 hours (Panel **B**). Nuclear extracts were prepared from treated cells and subjected to immunoprecipitations (IP) with an anti-CDK9 antibody or the control IgG. The complexes were immunodetected for the presence of CycT1 and HEXIM-1 by Western blotting (right panels). Input controls for CDK9, CycT1 and HEXIM1 are presented (left panels). Levels of β-actin were measured to control protein loading. Panels **A** and **B**. Histograms represent quantification of band intensities normalized to CDK9 levels in the IP and then normalized to mock-treated condition. Panel **C.** HeLa cells expressing YC.P-TEFb and VN.CTD were left untreated or were treated as indicated for 1 hour. Venus-positive cells were detected by fluorescence microscopy (upper panels). Bright-field images were also taken (lower panels). Panel **D.** HeLa cells expressing YC.P-TEFb and VN.CTD were treated as outlined in C and Venus-positive cells were counted and averaged from three different areas. Error bars represent differences between counts of Venus-positive cells from the randomly chosen fields under the microscope. Panel **E.** Hela cells were transfected with the Hex1(-104)Luc reporter plasmid. At 24 hours post-transfection, cells were mock-treated or treated with the different compounds as indicated. Luciferase activities in cell extracts were measured 24 hours after drug treatments and reported as fold increases over the activity observed in mock-treated condition (transfection of the reporter plasmid without drug treatment) and arbitrarily set at a value of 1. An experiment performed in duplicate representative of two independent experiments is shown.

To address the molecular mechanisms mediating the synergistic activation of HIV-1 transcription and production following the combined bryostatin-1+JQ1 treatment, we investigated the effect on P-TEB release of these LRAs alone or in combination. To this end, Jurkat cells were treated with JQ1, bryostatin-1 or a combination of both compounds for 1 hour and 24 hours. Nuclear extracts were prepared from treated cells and were used to perform immunoprecipitation experiments targeting CDK9 and Western blotting addressing the interaction of CDK9 with either HEXIM1 or CycT1. As shown in Fig 8A, for the 1 hour treatment, our results demonstrated a transient release of HEXIM1 from the CDK9/CycT1 (P-TEFb) complex after JQ1 treatment. These data are in agreement with a previous report from Peterlin and colleagues [31]. Bryostatin-1 treatment caused a weaker release of HEXIM1 from the P-TEFb complex than the JQ1 treatment (Fig 8A, compare lane 8 with lane 9). Interestingly, the combined treatment bryostatin-1+JQ1 led to a much stronger and synergistic HEXIM1 release than the individual treatments. These results indicated that that combined treatment increased the global availability of the active form of P-TEFb. After 24 hours of treatment (Fig 8B), we observed no P-TEFb release but a potentiated interaction between HEXIM1 and P-TEFb. This 24 hour effect contrasted with the 1 hour short-term effect and indicated reassembly of P-TEFb in the inactive 7SK snRNP complex. These results are consistent with the study of Liu *et al*. reporting that transient release of P-TEFb results in upregulation of its immediate target gene, HEXIM1, and reincorporation of P-TEFb into the 7SK snRNP [48]. Consistently, we also observed an increased expression of HEXIM1 after the 24 hours treatment compared to the 1 hour treatment (Fig 8B, input panel), thereby favoring a negative feedback mechanism of P-TEFb activation.

In order to study the effect of the PKC agonist+BETi/HMBA combined treatments on P-TEFb activation in living cells, we took advantage of the first and very elegant experimental system allowing to monitor quantitatively *in vivo* the interaction between P-TEFb and its substrate, the C-terminal domain (CTD) of RNA polymerase II (RNAPII). This system named V-PAC (visualization of P-TEFb activation by fluorescent complementation) [49] is based on a bimolecular fluorescence complementation (BiFC) assay and uses complementary fragments of fluorescent proteins, the N-terminal region (termed VN) of Venus and the C-terminal region (termed YC) of YFP fluorescent protein. Because activated P-TEFb interacts with and phosphorylates the RNAPII CTD, we employed P-TEFb and the CTD as fusion partners of YC and VN, respectively as described in [49]. Our results demonstrated that the number of YFP-positive cells was higher following the combined bryostatin-1+JQ1 treatment than the numbers obtained after the individual drug treatments (Fig 8C and 8D). Altogether, these data strongly indicated that combined LAR treatments led to higher activations of P-TEFb than the corresponding individual drug treatments.

To get further insights into the molecular mechanisms underlying the PKC agonist+BETi/HMBA synergistic gene expression activations induced by the combined treatments, we compared the effect of the drugs alone and in combination on P-TEFb-dependent transcription by reporter assays. More specifically, we transiently transfected HeLa cells with a reporter gene construct Hex1(-104)Luc containing the luciferase gene under the control of the HEXIM1 promoter which is responsive to active P-TEFb [48]. Transfected cells were mock-treated or treated with either PKC agonists (prostratin, bryostatin-1 and ing-B), either BETi (JQ1, I-BET and I-BET151), or HMBA individually and in combination (Fig 8E). Our results demonstrated synergistic increases in luciferase activity following combined treatments. Of note, the PKC agonist+JQ1 combinations were more potent than the other combinations tested.

**Fig 9:**
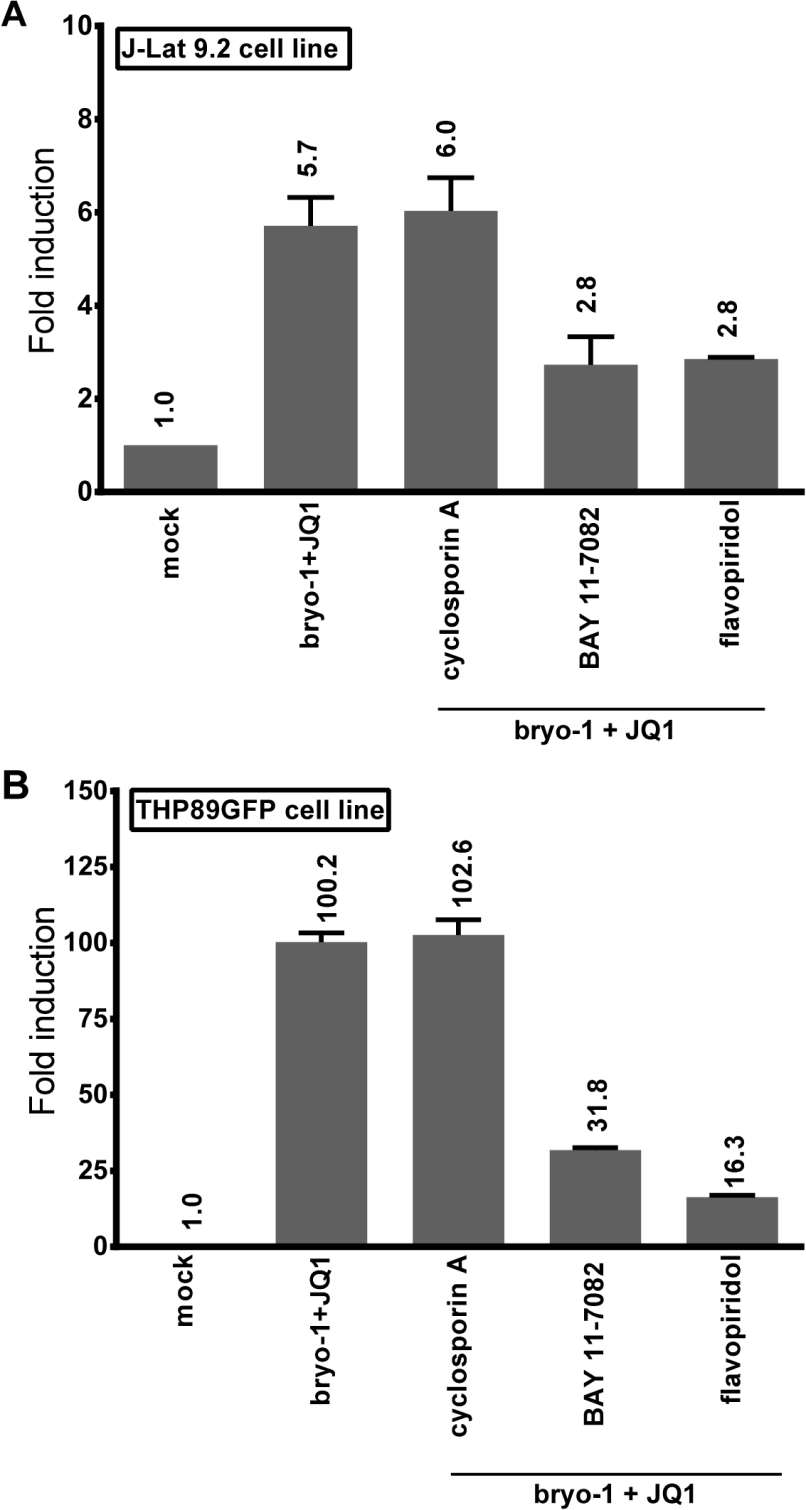
Bryostatin-1+JQ1-mediated HIV-1 reactivation is dependent on NF-κB and P-TEFb and independent from NFAT. The T-lymphoid J-Lat 9.2 (panel **A**) and monocytic THP89GFP (panel **B**) cell lines harbor latent HIV-1 provirus containing *gfp* gene. The cells were pre-treated with the indicated inhibitors for 2 hours and then either mock-treated or treated with the combination bryostatin-1 [10nM]+JQ1 [0.5μM]. At 24 hours post-treatment, cells were analyzed by flow cytometry to quantify the proportion of cells expressing GFP. Means and standard errors of the means from duplicate samples are indicated. One representative experiment from two is represented.

To further evaluate the signaling pathways leading to HIV-1 synergistic activation in response to PKC agonist+BETi cotreatments, we used chemical inhibitors targeting the NF-κB, P-TEFb and NFAT pathways in both T-lymphoid and monocytic infected cells lines (Fig 9A and 9B, respectively). We showed that BAY 11–7082, an inhibitor of IκBalpha phosphorylation, blocked an average of 50% and of 70% of the viral reactivation mediated by the combined treatment bryostatin-1+JQ1 in J-Lat 9.2 and THP89GFP cells, respectively. We used flavopiridol, a selective P-TEFb inhibitor, and we observed that this compound blocked an average of 50% and of 84% of viral activation mediated by the combined treatment bryostatin-1+JQ1 in J-Lat 9.2 and THP89GFP cells, respectively. In contrast, cyclosporin A, a potent NFAT inhibitor, had no effect on HIV-1 activation in both cell lines. These data confirmed that NF-κB and P-TEFb, as opposed to NFAT, were involved in induction of HIV-1 in response to bryostatin-1+JQ1 cotreatment.

Mechanistically, using four independent assays (immunoprecipitations, biomolecular fluorescence complementation assays, reporter gene assays and experiments using various signaling pathways inhibitors), we demonstrated that PKC agonists (such as bryostatin-1) and BETi (such as JQ1) caused a more potent activation of P-TEFb when used in combination than when used alone. Taken together, these mechanistic data established a correlation between the potentiated P-TEFb activation and the potentiated or synergistic (depending on the HIV-1 latency cellular model used) induction of HIV-1 gene expression observed after the combined versus individual drug treatments. This potentiated release of P-TEFb from the inactive 7SK snRNP complex could explain the potentiated or synergistic activation of HIV-1 gene expression induced by PKC agonist+BETi/HMBA combined treatments.

### PKC agonist+JQ1 co-treatments cause differential expression levels of cell surface activation markers and downregulate the expression of the CD4 receptor on CD4^+^ primary cells

**Fig 10:**
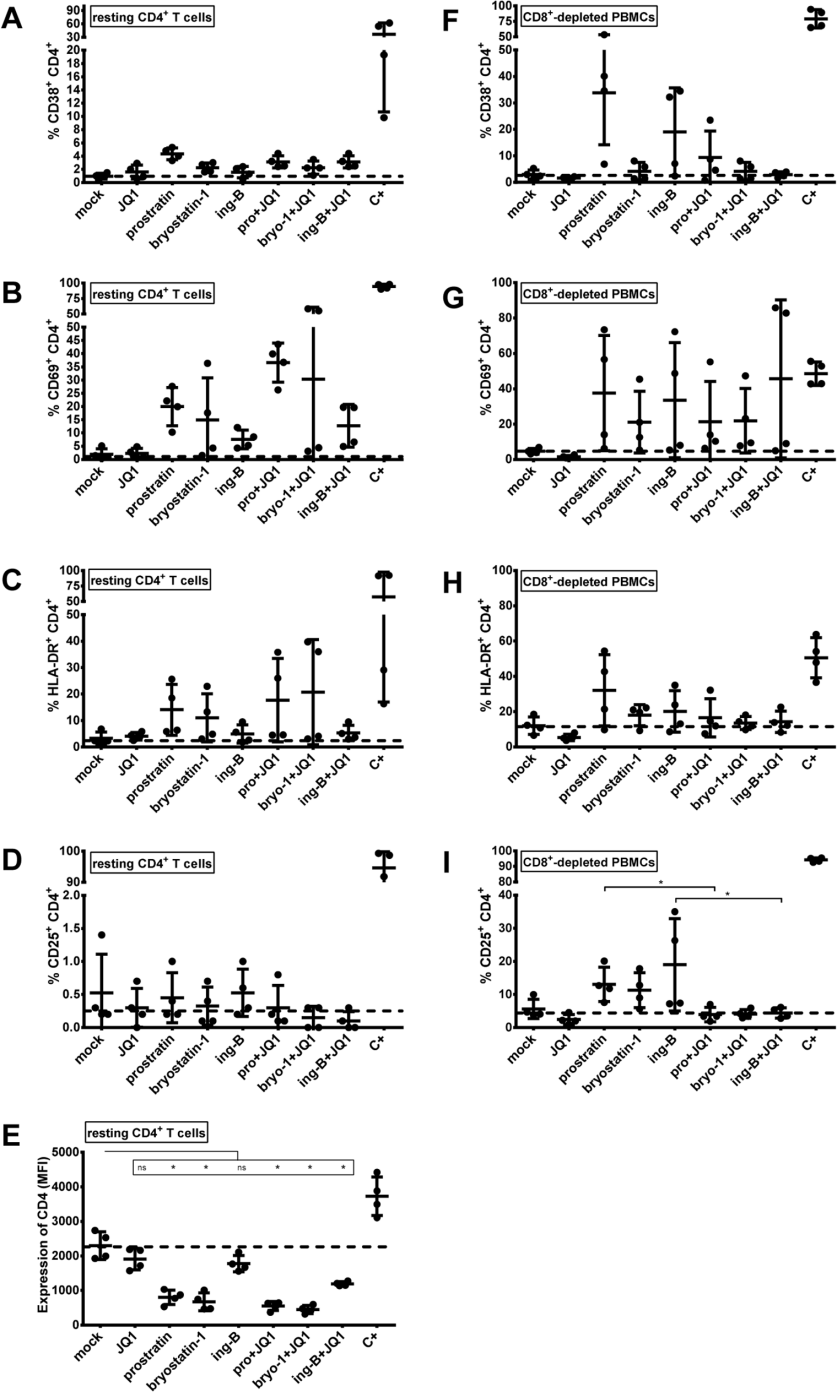
Expression of cell surface activation markers following PKC agonists and JQ1 treatments. Blood from 4 uninfected donors was split and one half was used to purify resting CD4^+^ T cells (panels **A**-**E**) and the other half was used to purify CD8^+^-depleted PBMCs (panels **F**-**I**). Cell cultures were mock-treated, treated with anti-CD3+anti-CD28 antibodies (C+), JQ1 [0.25μM], bryostatin-1 [5nM], prostratin [0.5μM] or ing-B [10nM] alone or in combination for 6 days. Cells were incubated with anti-CD38 (panels **A** and **F**), anti-CD69 (panels **B** and **G**), anti-HLA-DR (panels C and H), anti-CD25 (panels **D** and **I**) or anti-CD4 (panel **E**) antibodies prior to flow cytometry analysis. The results are presented as percentage of marker expression in the population of CD4^+^ cells (panels **A-D** and panels **F-I**) and as median fluorescence intensity (MFI) of CD4^+^ cells (panel **E**). Dashed line indicates the percentage of expression obtained in mock-treated cells. The means are represented. Statistical comparisons are indicated. Statistically relevant and not statistically relevant comparisons are indicated by asterisk and “ns”, respectively.

Compounds that would be suitable for the therapeutic use *in vivo* should not lead to non-specific or robust immune activation. Additionally, drug-mediated decrease of CD4 receptor surface expression may be an important factor in the blockade of *de novo* HIV-1 infection. To determine whether individual and combined treatments of PKC agonists with JQ1 led to the cellular activation, we isolated CD8^+^-depleted PBMCs and resting CD4^+^ T cells from blood of 4 uninfected donors. The activation status of resting CD4^+^ T cells and of CD4^+^ cells analyzed from the mock-treated or treated CD8^+^-depleted PBMCs population was assessed by flow cytometry analysis of the cell surface activation markers CD69, CD25, HLA-DR and CD38. First, we assessed the activation status of resting CD4^+^ T cells. We observed that JQ1 treatment did not cause any upregulation of T cell activation markers (Fig 10A–10D). Bryostatin-1 and prostratin alone or combined with JQ1 induced the surface expression of CD69 in 10–40% of cells and the surface expression of HLA-DR in 10–20% of cells (Fig 10B and 10C, respectively). These treatments led to low changes in the expression of CD38 and CD25 (Fig 10A and 10D, respectively). Ing-B alone or combined with JQ1 induced CD69 surface expression in 10–15% of cells (Fig 10B). Interestingly, there was no ing-B-induced modulation of other activation markers (Fig 10A, 10C and 10D). In order to extend the data we obtained in resting CD4^+^ T cells, we examined the expression levels of activation markers on the surface of CD4^+^ cells analyzed from the mock-treated or treated CD8^+^-depleted PBMCs population. Similarly to what we demonstrated in resting CD4^+^ T cells, we observed that JQ1 individual treatment did not cause any upregulation of activation markers (Fig 10F–10I). We showed that the individual and combined PKC agonists+JQ1 treatments led to differential increases in CD69, CD25, HLA-DR and CD38 surface expression levels. Interestingly, the combined treatments PKC agonists+JQ1 appeared to decrease PKC agonist-driven surface expression increases of CD38, HLA-DR and CD25 (Fig 10F, 10H and 10I, respectively). Since the CD38 marker is also constitutively expressed by naive CD4^+^ T cells, we analyzed the frequency of cells expressing simultaneously CD38 and HLA-DR (S7 Fig). Our data showed that the combined treatments PKC agonist+JQ1 appeared to decrease the PKC agonist-driven surface expression increases of these two activation markers. Next, we assessed the expression of CD4 surface receptor in resting CD4^+^ T cells following treatments with the drugs of interest (Fig 10E). PKC agonists were previously shown to exhibit anti-viral activity by decreasing surface expression of CD4 receptor which would lead to the blockade of *de novo* infection [16,22,25]. As shown in Fig 10E, we demonstrated that that bryostatin-1, prostratin and ing-B strongly reduced the cell surface expression of CD4 receptor. Individual JQ1 treatment had a slight effect on the CD4 surface expression and combined treatments of PKC agonis+JQ1 appeared to slightly augment the PKC agonist-driven CD4 receptor downregulation.

In conclusion, we showed that JQ1 caused no activation of resting CD4^+^ T cells and CD4^+^ cells analyzed from the treated CD8^+^-depleted PBMCs population. Combined treatments including JQ1 and PKC agonists caused elevated surface expression levels of CD69 and HLA-DR and no or low upregulation of CD38 and CD25. Notably, the decrease in surface expression levels of activation markers we observed in CD4^+^ cells from PBMCs following combined treatments as compared to the PKC agonists individual treatments strengthened the potential of PKC agonist+JQ1 co-treatments for purging strategies because it indicated that the latter combinations decreased the levels of global T-cell activation induced by PKC agonists. Moreover, the combinations PKC agonist+JQ1 led to downregulation of CD4 receptor, which could be beneficial for the blockade of *de novo* infection.

## Discussion

In this report, we performed an *in vitro* and *ex vivo* in-depth comparison of the reactivation potentials of two classes of latency-reversing agents, P-TEFb-releasing agents and PKC agonists. We demonstrated that combinations of LRAs from both classes led to strong synergistic activation of HIV-1 production in various post-integration latency cell line models of T-lymphoid and monocytic origins. Most importantly, we identified for the first time two potent combinations of LRAs, bryostatin-1+JQ1 and ing-B+JQ1, which activated latent HIV-1 to a degree comparable to that obtained with the positive control anti-CD3+anti-CD28 antibodies in a high number of *ex vivo* patient cell cultures and at multiple time points. Mechanistically, we provide a molecular explanation for these synergistic or potentiated viral reactivations by demonstrating a stronger activation of both P-TEFb and NF-κB activities following combined versus individual LRA treatments. We showed by bimolecular fluorescence complementation assays that the potent LRA effects observed resulted from a higher activation of P-TEFb. JQ1 alone failed to potently reactivate HIV-1 from latently-infected T cells. This could be explained by the fact that the level of P-TEFb is very low in resting T cells due to actions of specific miRNAs and the cellular factor NF-90 which block translation of CycT1 mRNA [50–53]. However, we demonstrated that JQ1 was a good candidate for combinatory treatments with compounds that increase the amount of P-TEFb.

The combined effects of PKC agonists and BET inhibitors are the result of the induction of P-TEFb production by PKC agonists [27,38,39] and of the release of P-TEFb from the repressive 7SK snRNP complex by BETi/HMBA [31,37]. In addition, Peterlin’s group has previously shown that PKC agonists also phosphorylate HEXIM1 at a specific serine residue, leading to release of active P-TEFb from the 7SK snRNP complex and induction of P-TEFb-dependent transcription [40]. In the present study, we confirmed this additional effect of bryostatin-1 and we demonstrated that the combination bryostatin-1+JQ1 caused a higher release of PTEF-b from its inactive 7SK snRNP complex than the drugs alone. We demonstrated a higher activation of P-TEFb following combined treatments of PKC agonist+BETi/HMBA by V-PAC assays. This higher P-TEFb activation can be explained by the dual effect of PKC agonists on P-TEFb (the CycT1 and CDK9 syntheses and the P-TEFb release from the 7SK snRNP complex) and by the additional effect of BETi/HMBA on the release of P-TEFb.

Moreover, McNamara and colleagues showed that, during activation of the inflammatory transcriptional program, the inducible recruitment of P-TEFb by NF-κB through PPM1G to promoters of inflammatory-responsive genes disassembles the 7SK snRNP and releases P-TEFb for transcription elongation [54]. Such an interaction could occur at the HIV-1 promoter, thereby providing an additional explanation for the synergistic activation of HIV-1 gene expression in response to PKC agonist+P-TEFb-releasing agent combined treatments.

Interestingly, our transient transfection experiments showed that combined treatments induced HIV-1 promoter-driven luciferase activity and that maximal synergistic activations required intact NF-κB binding sites in the enhancer region of the HIV-1 LTR. We showed that JQ1 synergistically increased bryostatin-1-induced NF-κB DNA-binding activity by EMSAs. This observation indicated that JQ1 played a role in controlling NF-κB activity either by targeting P-TEFb or by still unidentified mechanism(s). Therefore, this increase of NF-κB binding activity following the bryostatin-1+JQ1 treatment could be explained by the stabilization of the NF-κB/PPM1G/P-TEFb complex at the viral promoter. Recently, Zou and colleagues have shown that the BRD4 protein interacts with acetylated NF-κB and that this interaction maintains NF-κB constitutively active [55]. These authors have also demonstrated that JQ1 treatment disrupts this BRD4-acetylated NF-κB protein complex, leading to a decreased NF-κB transcriptional activity [42]. In contrast, the present study demonstrated that combined treatments PKC agonists+JQ1 exhibited beneficial effects on NF-κB activity (Fig 7A) and DNA-binding (Fig 7B). Additional studies will be necessary to further elucidate the role of JQ1 in NF-κB regulation.

Of note, the molecular mechanisms mediating the PKC agonists+BETi/HMBA synergy are likely to be highly complex and to implicate phenomena other than the P-TEFb and NF-κB regulations. For example, the following elements could also intervene in the molecular mechanisms of synergistic activation after combined PKC agonist+BETi/HMBA treatment: (i) NF- κB interactions with histone acetyltransferase, including p300 and CREB-binding protein (CBP) [56], (ii) direct interaction of P-TEFb with the cellular cofactor CTIP2 (COUP-TF-interacting protein 2) previously reported by our laboratory as involved in HIV-1 silencing in microglial cells [57] as well as (iii) the involvement of HMGA-1 (non-histone chromatin protein high mobility group AT-hook 1) in this P-TEFb/CTIP2 regulation [58].

Remarkably, Weinberger’s group has recently observed that conventional transcriptional activators synergize with “noise” modulators that affect gene expression fluctuations without changing mean expression [59]. Our data constitutes a good example of such a regulation since we observed a synergy between PKC agonists which represent conventional transcriptional activators and P-TEFb-targeting compounds that act at the level of transcriptional elongation (noise-enhancing compounds). Indeed, PKC agonists activate the LTR by increasing transcriptional initiation, thereby increasing “burst frequency”. This effect on transcriptional initiation is potentiated by the effect of compounds releasing P-TEFb (BETi/HMBA and PKC agonists themselves) that augment elongation duration, thereby increasing the transcriptional “burst size”.

Viral reactivation trials using LRA have been carried out and failed to demonstrate a reduction of the reservoirs size [60,61]. The important multicentric study conducted by Vincente Planelles and colleagues has shown that LRA often fail to reactivate HIV uniformly across different latency models [44]. The activity of a given LRA, demonstrated in a particular cellular model, cannot reliably predict its activity in other HIV-1 latency model systems. Therefore, it is essential to accumulate evidence for the reactivation potential of a single compound or combinations of compounds in several *in vitro* as well as *ex vivo* patient cell cultures in order to identify and validate potential promising reactivating treatments. In the present study, we first demonstrated the ability of co-treatments of PKC agonist+compound releasing active P-TEFb to activate expression of latent HIV *in vitro* not only in an HIV-1 T-lymphoid post-integration latency cell line model but also in promyelocytic, promonocytic, monocytic, and microglial latency cell lines. Indeed, several previous studies have reported that latency can also be established in cells of the monocyte/macrophage lineage including microglial cells, the brain resident macrophages and it is probably impossible to achieve an HIV cure without also considering these latent reservoirs (reviewed in [62]). We next confirmed the physiological relevance of the PKC agonist+BETi/HMBA co-treatments in *ex vivo* cultures of CD8^+^-depleted PBMCs or of resting CD4^+^ T cells isolated from cART-treated HIV-1^+^ aviremic patients. Spina *et al*.[44] have compared a panel of known stimuli *in vitro* in multiple cell model systems and *ex vivo* in resting CD4^+^ T cells from aviremic patients and they have reported that PKC agonists and PHA reactivate latent HIV uniformly across the different models, although drugs of other classes do not. Recently, Siliciano and colleagues have demonstrated that among a panel of LRA, only bryostatin-1 effectively reversed HIV-l latency. Importantly, the ability of bryostatin-1 to induce HIV-1 recovery was much lower than the effect obtained with the positive control in the three independent *ex vivo* assays used by these authors [63]. Here, we demonstrated that P-TEFb releasing compounds, especially JQ1, potentiated the effect of PKC agonists. Remarkably, the mean extracellular HIV-1 RNA level obtained with the bryostatin-1+JQ1 combination was similar to the mean level obtained with the positive control. Moreover, the percentage of reactivated cultures following treatment with this combination was identical to the percentage we observed with the positive control (79%). We confirmed in resting CD4^+^ T cells isolated from 15 cART-treated HIV^+^ patients the results obtained in the *ex vivo* cultures of CD8^+^-depleted PBMCs. Indeed, we observed that the combinations of JQ1 with bryostatin-1 or with another promising PKC agonist, ing-B, activated latent HIV-1 to a mean extracellular HIV-1 RNA level similar to that obtained with the anti-CD3+anti-CD28 antibodies positive control stimulation. This result constitutes the first demonstration of anti-latency combinations exhibiting such a potent effect. Importantly, we confirmed in the presence of cART the HIV reactivation potentials of the two most promising combinations *ex vivo* and furthermore demonstrated that their potent effects were already detected 24 hours post-treatment. Indeed, not only at 24 hours but also at 72 hours post-stimulation, the percentage of reactivated cultures following treatment with the two combinations was higher than the percentage we observed with the positive control. Surprisingly, the effect of the bryostatin-1+JQ1 cotreatment was statistically superior to that observed after a 24 hours global T cell activation. This latter observation would be of interest in the perspective of a clinical trial since bryostatin-1 is generally administrated as continuous infusion of 24 hours [46] or 72 hours [47].

It should be noted that we did not observe viral reactivation either after individual, combined or anti-CD3+anti-CD28 treatments in all patient cell cultures, thereby supporting the notion that establishment of viral latency is a multifactorial and stochastic phenomenon. Siliciano’s group has recently demonstrated in an elegant study that only some replication-competent viruses could be activated after single round of maximum *in vitro* stimulation [64]. Therefore, multiple rounds of stimulation might be necessary in order to reach efficient HIV-1 reactivation. Moreover, as reported by us previously [65,66], we observed here again high diversity among the patient cell cultures in terms of pattern of response to the different LRAs tested. This phenomenon reflects the heterogeneity of the reservoirs and the multiplicity of the mechanisms which underlie latency and probably vary from one patient to the other and even from one cell to the other in single patient. This observation highlights the necessity to test a great number of patient blood samples in *ex vivo* experiments in order to identify promising LRA. It also emphasizes the need to evaluate the efficacy of an LRA first *ex vivo* in cell cultures from a given patient before the administration of this LRA to this given patient *in vivo* in the context of a clinical trial.

Among the characteristics necessary for clinically testable LRAs, toxicity is a crucial concern. The major problem with PKC activators is the nonspecific induction of many genes following activation of PKC-related pathways and the toxicity caused by the systematic release of cytokines. Indeed, an ideal LRA for a shock and kill strategy should reverse HIV from latency without causing a broad and robust T-cell activation [15]. Nevertheless, low level and short-term T-cell activation will likely be required for efficient HIV-1 reactivation since viral transcription is directly affected by events related to cellular activation including translocation of the NF-κB transcription factor. Combinations of either bryostatin-1 or ing-B with JQ1 and the synergistic effects obtained here *in vitro* and *ex vivo* with these molecules allowed us to use very low drug concentrations and could therefore cause *in vivo* lower toxic effects, notably due to global T-cell activation. Importantly, our data strengthened the advantage of combined treatments not only because of reduced drug concentrations but also because the PKC agonist+JQ1 combined treatments appeared to further decrease the PKC agonist-driven elevated surface expression levels of activation markers. PKC agonists have been previously reported to exhibit anti-viral activity by decreasing surface expression of CD4 receptor that could favor the blockade of *de novo* infection [67]. Interestingly, in the present study, we observed that combinations of PKC agonists with JQ1 tended to even augment the PKC agonist-driven downregulation of CD4 receptor.

Finally, our in-depth comparison of PKC agonist+compound releasing P-TEFb co-treatments allowed us to identify the bryostatin-1+JQ1 and ing-B+JQ1 combinations as presenting a potent ability to induce expression of latent HIV. Bryostatin-1 is of particular interest because it has been tested in more than 20 clinical trials for cancer and Alzheimer’s disease. Importantly, a phase I clinical trial with bryostatin-1 has been performed in children with solid tumors and peak concentrations of bryostatin-1 (10–20x10^-9^ M) reached in several patients were higher than the concentration we used in our *ex vivo* experiments (5nM) [68]. The bryostatin-1 infusion has been well tolerated in this phase I clinical trial since only few patients have experienced myalgia, photophobia or eye pain [53]. Moreover, ing-B has been evaluated in *vivo* in rats, dogs, and Rhesus macaques by oral dosing and presents low toxic profiles (Aurigon Life Science, and L. Gama, oral communication). Of note, another ingenol ester, called ingenol-3-angelate, is used for treatment of actinic keratosis [69]. However, ingenol-3-angelate is only used as a topical cutaneous treatment and is practically not absorbed through the skin (http://www.drugs.com/pro/picato.html). Importantly, ingenol-3-angelate appears to be more toxic than ing-B when orally delivered to rats and dogs (Luiz Pianowski, Kyolab, Brazil, personal communication). Regarding the BETi, JQ1 presents very promising anti-cancer effects in preclinical trials [70,71] as well as anti-inflammatory effects [72]. Clinical trials with JQ1-derivative called TEN-010 (also called JQ2) and another BETi called GSK525762 have been initiated recently to characterize their safety, tolerability, pharmacokinetics and anti-cancer activity (clinicaltrials.gov).

Nevertheless, we are aware that the fate of pharmacologically reactivated HIV^+^ cells is not fully understood. Even a potent activation of HIV-1 expression might not be sufficient enough to kill the reactivated cells and to decrease the size of the latent reservoirs. Additional interventions would be needed to eliminate cells expressing viral proteins such as immunostimulatory strategies to enhance antiviral immune effector functions [for example by boosting cytotoxic T lymphocytes (CTL) responses] [73]. In conclusion, we reported a proof-of-concept for the co-administration of two different types of LRAs (a PKC agonist and a compound releasing P-TEFb) as a therapeutic perspective to decrease the size of the HIV-1 reservoirs in presence of efficient cART. This study not only identifies new promising LRAs combinations but also highlights the patient-specific variations in the responses to these HIV-1 inducers, likely reflecting heterogeneity in the molecular mechanisms regulating HIV-1 latency. The understanding of the factors involved in this patient-to-patient diversity represents a key challenge in the race for a cure or a durable remission of HIV infection.

Of note, during the preparation of our manuscript, Siliciano group has reported that protein kinase C agonists in combination with JQ1 or HDACi robustly induce HIV-1 transcription and production in cell cultures from blood of cART-treated HIV^+^ patients [74].

## Materials and Methods

### Cell lines and cell culture

The CD4^+^ T-lymphoid cell lines Jurkat, J-Lat 9.2, J-Lat A2 (harboring an LTR-Tat-IRES-GFP construct), J-Lat A72 (harboring LTR-GFP construct) and myeloid lineage cell lines U1 and OM10.1 were obtained from the AIDS Research and Reference Reagent Program (National Institute of Allergy and Infectious Diseases [NIAID], National Institute of Health [NIH]). The monocytic THP89GFP cell line [42] is a kind gift from David N. Levy (New York University College of Dentistry, New York, USA). These cell lines were cultured in RPMI 1640 medium (Gibco-BRL) supplemented with 10% fetal bovine serum, 50 U/ml of penicillin, 50 mg/ml of streptomycin at 37°C in a humidified 95% air/5% CO_2_ atmosphere. The microglial cell line CHME-5/HIV [41] is a kind gift from Jonathan Karn (Case Western Reserve University, Cleveland, Ohio, USA). This cell line was cultured in Dulbecco’s modified Eagle’s-Glutamax I medium (Invitrogen) supplemented with 10% fetal bovine serum, 50 U/ml of penicillin, 50 mg/ml of streptomycin at 37°C in humidified 95% air/5% CO_2_ atmosphere.

### Reagents

TNFα (300-01A) was purchased from Immunosource. Prostratin (12 deoxyphorbol-13-acetate) (PE 187–0001) were provided by Enzo Life Sciences. Bryostatin-1 (B7431) was purchased from Sigma. Ing-B was kindly donated by Luiz F. Pianowski, Kyolab/Amazônia Fitomedicamentos, Valinhos, Sao Paulo, Brazil. JQ1 (2091–1) and I-BET151 (2220–1) were purchased from BioVision. I-BET (401010) was purchased from Calbiochem. HMBA (H4663) was purchased from Sigma. Human CD3 (IMI1304) and CD28 (IMI1376) antibodies were obtained from Analis. BAY 11–7082 (B5556), Flavopiridol (F3055) and cyclosporin A (C2163000) were purchased from Sigma. Efavirenz (4624), Zidovidine (3485) and Raltegravir (11680) were obtained from the AIDS Research and Reference Reagent Program (National Institute of Allergy and Infectious Diseases [NIAID], National Institute of Health [NIH]).

All compounds, resuspended and stored as recommended by the manufacturer, were diluted immediately before use in cell culture medium.

### Cellular metabolic activity and viability assays

Cellular metabolic activity was evaluated by the colorimetric test WST-1 (Roche). Cell viability was assessed by trypan blue exclusion assay.

### Virus production assays

HIV-1 production was measured in the supernatant of the J-Lat 9.2, U1 and OM10.1 cell cultures by determining CA-p24 antigen ELISA (FUJIREBIO).

### Flow cytometry

J-Lat 9.2, J-Lat A2, J-Lat A72, THP89GFP and CHME-5/HIVcell lines were mock-treated or treated for 24 hours with the different compounds alone or in combination. Cells were washed twice in PBS, resuspended in PBS containing 4% paraformaldehyde and fixed for 1 hour in the dark. Cells were next washed twice in PBS and resuspended in FACS buffer (PBS, BSA 0.1%, NaN3 0.1%). The percentage of GFP-positive cells was measured on a CXP cytometer (Cytomics FC 500, Beckman Coulter) using CXP Software version 1.0 according to the manufacturer’s instructions.

### RNA extractions and analyses of initiated and elongated HIV-1 transcripts

Total RNA samples were isolated using the RNeasy Plus kit (Qiagen) from 1x10^6^ cells and digested with TURBO DNase (TURBO DNA-freeTM kit, Ambion). First strand cDNA was synthesized using SuperScript III Reverse Transcriptase (Invitrogen). Quantitative real-time PCR reactions were performed using the PerFecTa SYBR Green Super Mix, ROX (Quanta). Initiated transcripts were detected with primers TAR (FW, 5’-GTTAGACCAGATCTGAGCCT- 3’ and RV, 5’-GTGGGTTCCCTAGTTAGCCA- 3’). Elongated transcripts were detected with two different sets of primers: *tat* (FW, 5’-ACTCGACAGAGGAGAGCAAG-3’ and RV 5’-GAGATCTGACTGTTCTGATGA-3’) and *env* (FW, 5’-GACATTTGTACATGGTCCTGTTC-3’ and RV, 5’- GGCTGGTTTTGCGATTCTAA-3’). cDNA were quantified and normalized to the β-Actin mRNA level as previously described [65].

### Transient transfection and luciferase reporter assays

Jurkat cells were transfected with the pLTR-luc and the pLTR-NFκBmut-luc luciferase reporter episomal vectors using JetPEI TM (POLYplus) method according to the manufacturer’s protocol. Twenty-four hours post-transfection, cells were mock-treated or treated with the different compounds as indicated. At 24 hours post-treatment, cells were lysed and assayed for luciferase activity (Promega) as previously described [66]. Luciferase activities were normalized with respect to protein concentrations.

HeLa cells were transiently transfected with the Hex1(-104)Luc reporter plasmid [48] by X-tremeGENE HP Transfection Reagents (Roche). At 24 hours post-transfection, cells were mock-treated or treated with the different compounds as indicated. At 24 hours post-treatment, cells were lysed and assayed for luciferase activity (Promega). Luciferase activities were normalized with respect to protein concentrations.

### V-PAC assay

V-PAC assays were performed as described previously with a minor modification by using more potent Venus fluorescent proteins instead of original Yellow Fluorescent proteins [49]. Briefly, 1 million of HeLa cells were transfected with 0.4μg of plasmid DNA encoding YC-P-TEFb and 1.2μg of plasmid DNA encoding VN-CTD using X-tremeGENE Transfection Reagents (Roche). Twenty-four hours post-transfection, the cells were split into 24-well plates and kept in 5% FCS. At 48 hours post-transfection, the cells were mock-treated or treated with the different compounds as indicated for 60 min. Fluorescence signals were detected by microscopic analysis using Olympus IX70 bright field fluorescent microscope. The fluorescence images were analyzed using Metamorph software and Venus positive cells were manually counted and averaged from three randomly chosen fields of each sample.

### EMSAs

Jurkat cells were mock-treated or treated with various compounds as indicated and 24 hours post-treatment cells were subjected to nuclear extract preparation using NE-PER Nuclear and Cytoplasmic Extraction Reagents (Thermo Scientific). EMSAs with the HIV-1 NF-κB probe were performed as previously described [65]. As loading controls, the same nuclear extracts were tested for binding of Oct-1 (octamer-binding protein-1) to an Oct-1 consensus probe as previously described [65].

### Coimmunoprecipitation assays and western blot analyses

Immunoprecipitations with anti-CDK9 (sc13130, Santa Cruz) antibody and control IgG (Millipore) were performed with 100μg of nuclear extracts from Jurkat cells. The immunoprecipitated proteins were processed for SDS/PAGE and Western blot analysis. The P-TEFb complexes were detected using antibodies directed against CycT1 (sc8127), CDK9 and HEXIM1 (sc48872) (Santa Cruz). Western blotting of the inputs were performed with 20μg of the nuclear extracts. The β-actin (A5441, Sigma) was immunodetected as loading control. Quantifications of the CDK9-associated HEXIM1 and CycT1 were performed with the ImageJ software (National Institutes of Health, NIH).

### Study subjects

We selected 50 HIV-1-infected individuals at the St-Pierre Hospital (Brussels, Belgium) on the basis of the following criteria: all volunteers were treated with cART for at least 1 year, had an undetectable plasma HIV-1 RNA level (20 copies/ml) for at least 1 year and had a level of CD4^+^ T lymphocytes higher than 300 cells/mm^3^ of blood. Characteristics (age, CD4^+^ T cell count, CD4^+^ nadir, antiviral regimens, duration of therapy, duration with undetectable plasma HIV-1 RNA level, and HIV-1 subtypes) of patients from the St- Pierre Hospital were well documented and are presented in the S1 Table.

### Ethics statement

Ethical approval was granted by the Human Subject Ethics Committees of the Saint-Pierre Hospital (Brussels, Belgium). All individuals enrolled in the study provided written informed consent for donating blood.

### Isolation of CD8^+^-depleted PBMCs and resting CD4^+^ T cells

CD8^+^-depleted PBMCs used in reactivation assays were isolated from fresh whole blood of HIV^+^ patients as previously described [65] [66]. Resting CD4^+^ T cells were isolated from PBMCs of HIV^+^ patients using a negative selection on a Robosep (Stemcell) using cocktail of antibodies (Custom kit, Stem Cells). For each treatment, six million CD8^+^-depleted PBMCs or 0.5 million of resting T cells (CD8^-^,CD14^-^, CD16^-^, CD19^-^, CD20^-^, CD36^-^, CD56^-^, CD66b^-^, CD123^-^, TRCγ/δ^-^, glycoprotein A^-^, HLA-DR^-^, CD69^-^, CD25^-^) were seeded in LGM-3 Growth Medium (Lonza). One day after isolation, cells were mock-treated or treated with anti-CD3/CD28 antibodies as a positive control or by various LRAs for 6 days. Medium was harvested at day 1, day 3 and day 6, replaced with fresh medium containing compounds when appropriate at day 1 and day 3 and used for quantification of HIV-1 RNA. For the time course experiment, 12 million of PBMCs were seeded in the presence of antiretrovirals [Efavirenz (100nM), Zidovudine (180nM), Raltegravir (200nM)].

### Quantification of total HIV-1 DNA

The total cellular DNA was extracted from patient CD8^+^-depleted PBMCs or resting CD4^+^ T cell *ex vivo* cultures using the QIA amp DNA Mini or the QIA amp Micro kit (Qiagen), respectively. The total cell-associated HIV-1 DNA was then quantified by ultra-sensitive real-time PCR (Generic HIV DNA cell kit, Biocentric [75]) according to the manufacturer’s instructions.

### Quantitative assessment of HIV-1 RNA from culture supernatants of patient cells

Six days after drug treatment, culture supernatants from either patient CD8^+^-depleted PBMCs or patient resting CD4^+^ T cell *ex vivo* cultures were collected for RNA extraction using QIA amp Viral RNA Mini kit (Qiagen). HIV-1 RNA levels were quantified using the Generic HIV Charge Virale kit (Biocentric) according to the manufacturer’s instructions (detection limits of 150 HIV-1 RNA copies/ml or 15 HIV-1 RNA copies/ml depending on tested supernatant volumes).

### Infection assays in Jurkat cell line with *ex vivo* reactivated viruses

The outgrowth viruses, previously quantified by the determination of extracellular HIV-1 RNA concentration, were concentrated by ultracentrifugation of 3ml of culture supernatants collected 6 days post-treatment and resuspended in 50μl of culture medium. Infections of Jurkat cells were performed by resuspending 50 thousand cells in 50μl of the outgrowth viral stocks and incubating at 37°C for 2 hours.The multiplicity of infection was in the range of 0.02–0.05. After infection, the cells were pelleted at 300xg, washed three times with 200μl of culture medium, resuspended in 200μl of culture medium, and grown under standard conditions in 96-well plates. Two days post-infection, cells were amplified by adding 400μl of culture medium and seeded in 24-well plates. Six days post-infection, 350μl of culture supernatants were collected for RNA extraction using QIAamp Viral Mini Kit (Quiagen) according to manufacturer’s instructions. HIV-1 RNA levels were quantified using the Generic HIV Charge Virale kit (Biocentric) (detection limit of 75 HIV-1 RNA copies/ml).

### Cell activation analysis by flow cytometry

For cell activation analysis, CD8^+^-depleted PBMCs or resting CD4^+^ T cells isolated from blood of uninfected donors were used to establish *ex vivo* culture cells. Cells were collected 6 days after stimulation with different LRAs and were stained with relevant antibodies. All antibodies were purchased from BD Biosciences and included three antibody cocktails. The first cocktail included anti-CD4 (557852), anti-CD8 (345774) and anti-CD38 (345806). The second cocktail included anti-CD4 (345770), anti-CD8 (348813), anti-HLA-DR (347401), anti-CD69 (347823) and anti-CD25 (340907). The third cocktail included anti-CD4 (345770), anti-CD8 (345772), anti-CD38 (555462) and anti-HLA-DR (347401). Marker staining was assessed by flow cytometry analysis on a FACS CantoII (Becton-Dickinson) and analyzed using the FACSDiva Software (Becton-Dickinson).

### Statistical analysis

T-cell activation data, *ex vivo* reactivation studies using patient cell cultures of CD8^+^-depleted PBMCs or of resting CD4^+^ T cells are shown as means. Data sets were analyzed using an unpaired, nonparametric Mann-Whitney U test. P<0.05 was considered statistically relevant. Analyses were performed using Prism version 6.0 (GraphPad software).

## Supporting Information

S1 Fig PKC agonists and compounds releasing active P-TEFb increase HIV-1 production in a dose-dependent manner with marginal effects on cell viability. The J-Lat 9.2 (panels **A, C, E**, and **G**) and U1 (panels **B, D, F**, and **H**) cell lines were mock-treated or treated with increasing doses of compounds as indicated. At 24 hours post-treatment, CA-p24 production in cell supernatants (panels **A**, **B, E,** and **F**) or cell metabolic activity (panel **C**, **D**, **G**, and **H**) were measured. Results obtained with the mock-treated cells were arbitrary set at a value of 1 or 100%, respectively. Means and standard errors of the means from duplicate samples are indicated. One representative experiment from three is represented.
(PPT)

S2 Fig PKC agonists and compounds releasing active P-TEFb increase HIV-1 production in OM10.1 cell lines. OM10.1 cells were mock-treated or treated with JQ1 (0.5μM), I-BET (0.5μM), I-BET151 (0.5μM), HMBA (5Mm), bryostatin-1 (10nM) and prostratin (2.5 μM) alone or in combination as indicated. At 24 hours post-treatment, CA-p24 production in cell supernatants were measured. Results obtained with the mock-treated cells were arbitrary set at a value of 1 or 100%, respectively. Means and standard errors of the means from duplicate samples are indicated. One representative experiment from two is represented. For each combinatory treatment, the fold-synergy was calculated by dividing the effect observed after co-treatments by the sum of the effects after the individual treatments.
(PPT)

S3 Fig PKC agonist+BETi/HMBA combined treatments increase HIV-1 expression in a higher proportion of cells than the drug alone. The THP89GFP cells (panel **A**), J-Lat cell line A2 (containing stably integrated LTR-Tat-IRES-GFP construct, panel **B**) or A72 (panel **C**) containing a stably integrated LTR-GFP construct were mock-treated, treated with JQ1 (0.5μM), I-BET (0.5μM), I-BET151 (0.5μM), HMBA (5mM), bryostatin-1 (10nM) and prostratin (2.5 μM) alone or in combination as indicated. At 24 hours post-treatment, cells were analyzed by flow cytometry to quantify the proportion of cells expressing GFP. Means and standard errors of the means from duplicate samples are indicated. One representative experiment from two is represented. For each combinatory treatment, the fold-synergy was calculated by dividing the effect observed after co-treatments by the sum of the effects after the individual treatments.
(PPT)

S4 Fig PKC agonist+BETi/HMBA combined treatments increase the expression of GFP. The J-Lat 9.2 cell line (panel **A**), CHME-5/HIV microglial cells (panel **B**) or THP89GFP monocytic cells (panel **C**) harbor latent HIV1 provirus containing *gfp* gene. The cells were mock-treated, treated with JQ1 (0.5μM), I-BET (0.5μM), I-BET151 (0.5μM), HMBA (5mM), bryostatin-1 (10nM) and prostratin (2.5 μM) alone or in combination as indicated. At 24 hours post-treatment, cells were analyzed by flow cytometry and the mean fluorescence intensity (MFI) was analyzed to quantify the amount of GFP produced. Means and standard errors of the means from duplicate samples are indicated. One representative experiment from three is represented. For each combinatory treatment, the fold-synergy was calculated by dividing the effect observed after co-treatments by the sum of the effects after the individual treatments.
(PPT)

S5 Fig Effects of BETi, HMBA and PKC agonists on cell viability in CD8^+^-depleted PBMCs. WST-1 assay on *ex vivo* cultures of CD8^+^-depleted PBMCs isolated from blood of 5uninfected donors were incubated with indicated compounds for 6 days. The result obtained with mock-treated cells was set at a value of 100%.
(PPT)

S6 Fig Effects of PKC agonists and JQ1 individual and combined treatments on cell viability in CD8^+^-depleted PBMCs. Panel **A.** WST-1 assay on *ex vivo* cultures of CD8^+^-depleted PBMCs isolated from blood of 4 uninfected donors were incubated with indicated compounds for 6 days. The result obtained with mock-treated cells was set at a value of 100%. Panel **B**. Cell viability. Trypan blue exclusion assay was performed on the same patient cell cultures as described in (A).The result obtained with mock-treated cells was set at a value of 100%.
(PPT)

S7 Fig Expression of the CD38 and the HLA-DR cell surface activation markers following PKC agonists and JQ1 treatments. CD8^+^-depleted PBMCs from 4 uninfected donors were mock-treated, treated with anti-CD3+anti-CD28 antibodies (C+), JQ1 (0.25μM), bryostatin-1 (5nM), prostratin (0.5μM) or ingenol B (10nM) alone or in combination for 6 days. Cells were incubated with anti-CD38, anti-HLA-DR, anti-CD4 and anti-CD8 antibodies prior to flow cytometry analysis. The results are presented as percentage of marker expression in the population of CD4^+^ cells. Dashed line indicates the percentage of expression obtained in mock-treated cells. The means are represented.
(PPT)

S1 Table Presentation of patient characteristics. Characteristics (age, CD4^+^T cell count, CD4^+^ nadir, antiviral regimens, duration of therapy, duration with undetectable plasma HIV-1 RNA level, and HIV-1 subtypes) of patients from the St- Pierre Hospital are presented. “X” indicates not reported.
(PPT)

S2 Table Infections of Jurkat cells with viruses isolated from bryostatin-1+JQ1-treated *ex vivo* patient cell cultures. *Ex vivo* cultures of CD8^+^-depleted PBMCs from blood of 3 cART-treated HIV^+^ patient were treated with bryostatin-1+JQ1 for 6 days. Concentrations of viral RNA in culture supernatants were measured and were expressed as HIV-1 RNA copies/ml. Total HIV-1 DNA was expressed as HIV-1 DNA copies/10^6^ CD8^+^-depleted PBMCs. Viruses collected from *ex vivo* cell cultures were used to perform *de novo* infection of Jurkat cells. Six days post-infection, Jurkat culture supernatants were collected and concentrations of viral RNA in culture supernatants were quantified and expressed as HIV-1 RNA copies/ml).
(PPT)
