# Supplementary figures and images for: An In-Depth Comparison of Latency-Reversing Agent Combinations in Various In Vitro and Ex Vivo HIV-1 Latency Models Identified Bryostatin-1+JQ1 and Ingenol-B+JQ1 to Potently Reactivate Viral Gene Expression
Source: PLoS Pathog. 2015 Jul 30;11(7):e1005063. doi: 10.1371/journal.ppat.1005063 (PMC4520688; doi:10.1371/journal.ppat.1005063)

## Slide 1
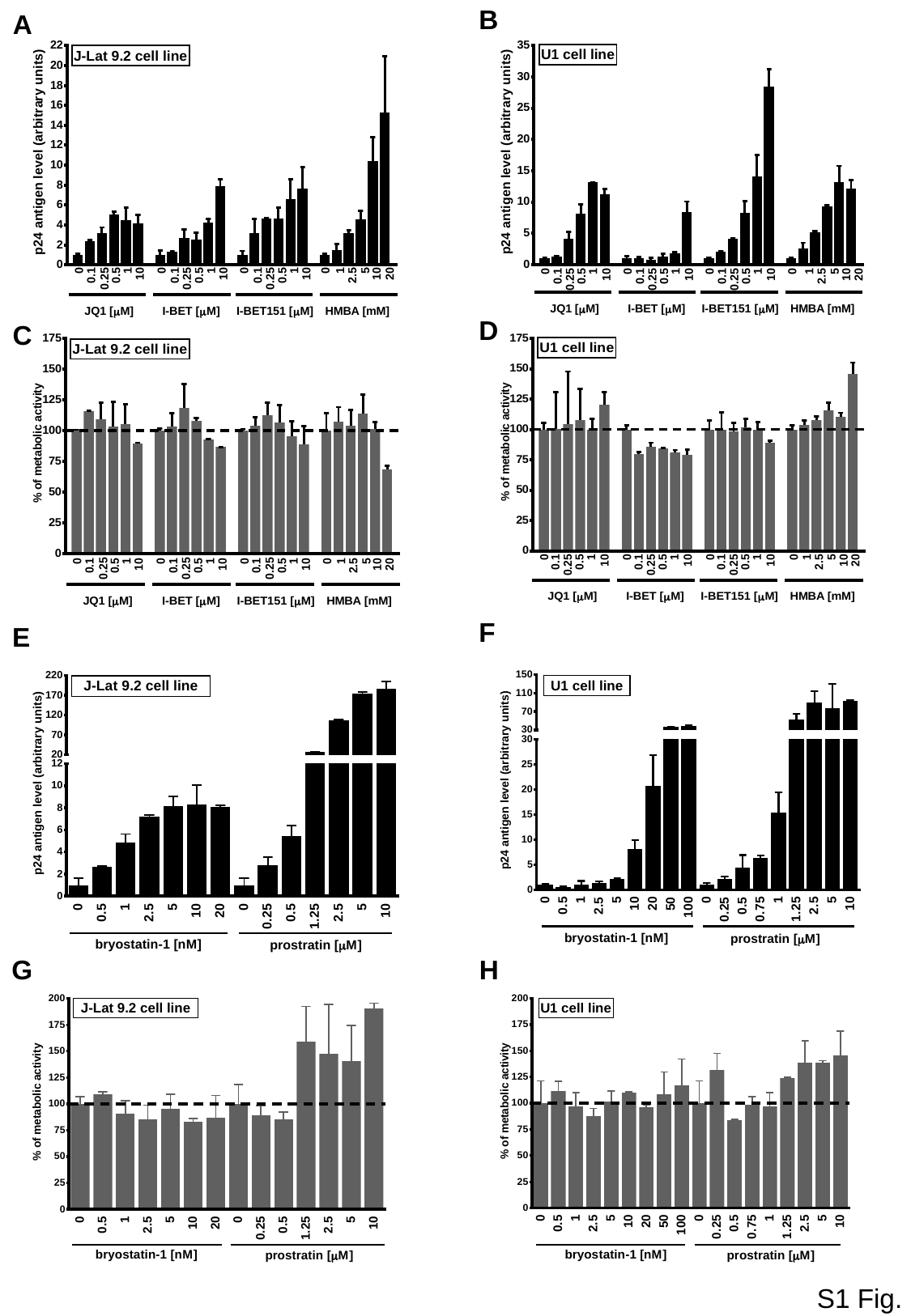

B
A
D
C
F
E
G
H
S1 Fig.

Supplement: S1 Fig — The J-Lat 9.2 (panels A, C, E, and G) and U1 (panels B, D, F, and H) cell lines were mock-treated or treated with increasing doses of compounds as indicated. At 24 hours post-treatment, CA-p24 production in cell supernatants (panels A, B, E, and F) or cell metabolic activity (panel C, D, G, and H) were measured. Results obtained with the mock-treated cells were arbitrary set at a value of 1 or 100%, respectively. Means and standard errors of the means from duplicate samples are indicated. One representative experiment from three is represented. (PPT) [file ppat.1005063.s001.ppt]

## Slide 1
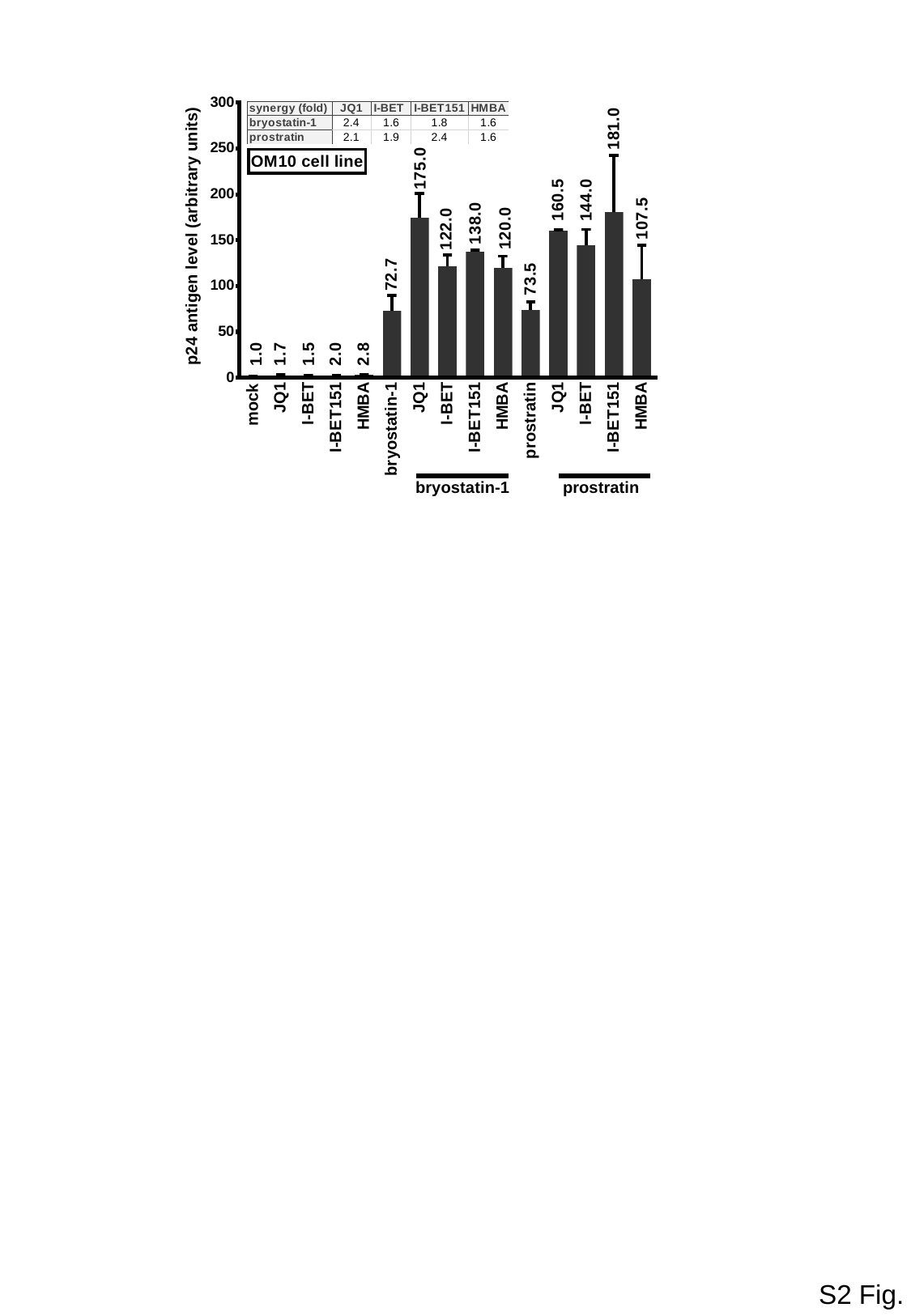

S2 Fig.

Supplement: S2 Fig — OM10.1 cells were mock-treated or treated with JQ1 (0.5μM), I-BET (0.5μM), I-BET151 (0.5μM), HMBA (5Mm), bryostatin-1 (10nM) and prostratin (2.5 μM) alone or in combination as indicated. At 24 hours post-treatment, CA-p24 production in cell supernatants were measured. Results obtained with the mock-treated cells were arbitrary set at a value of 1 or 100%, respectively. Means and standard errors of the means from duplicate samples are indicated. One representative experiment from two is represented. For each combinatory treatment, the fold-synergy was calculated by dividing the effect observed after co-treatments by the sum of the effects after the individual treatments. (PPT) [file ppat.1005063.s002.ppt]

## Slide 1
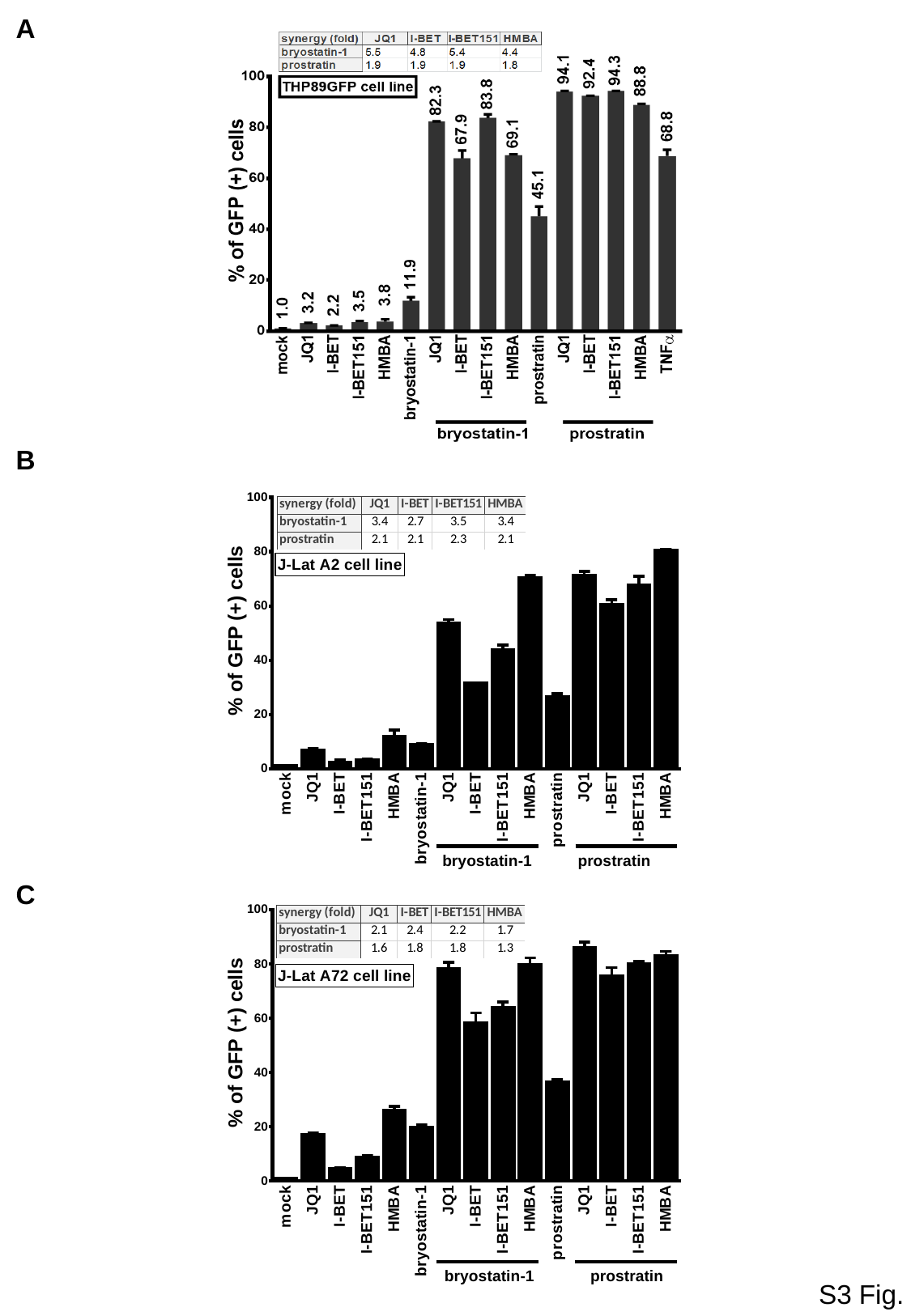

A
B
C
S3 Fig.

Supplement: S3 Fig — The THP89GFP cells (panel A), J-Lat cell line A2 (containing stably integrated LTR-Tat-IRES-GFP construct, panel B) or A72 (panel C) containing a stably integrated LTR-GFP construct were mock-treated, treated with JQ1 (0.5μM), I-BET (0.5μM), I-BET151 (0.5μM), HMBA (5mM), bryostatin-1 (10nM) and prostratin (2.5 μM) alone or in combination as indicated. At 24 hours post-treatment, cells were analyzed by flow cytometry to quantify the proportion of cells expressing GFP. Means and standard errors of the means from duplicate samples are indicated. One representative experiment from two is represented. For each combinatory treatment, the fold-synergy was calculated by dividing the effect observed after co-treatments by the sum of the effects after the individual treatments. (PPT) [file ppat.1005063.s003.ppt]

## Slide 1
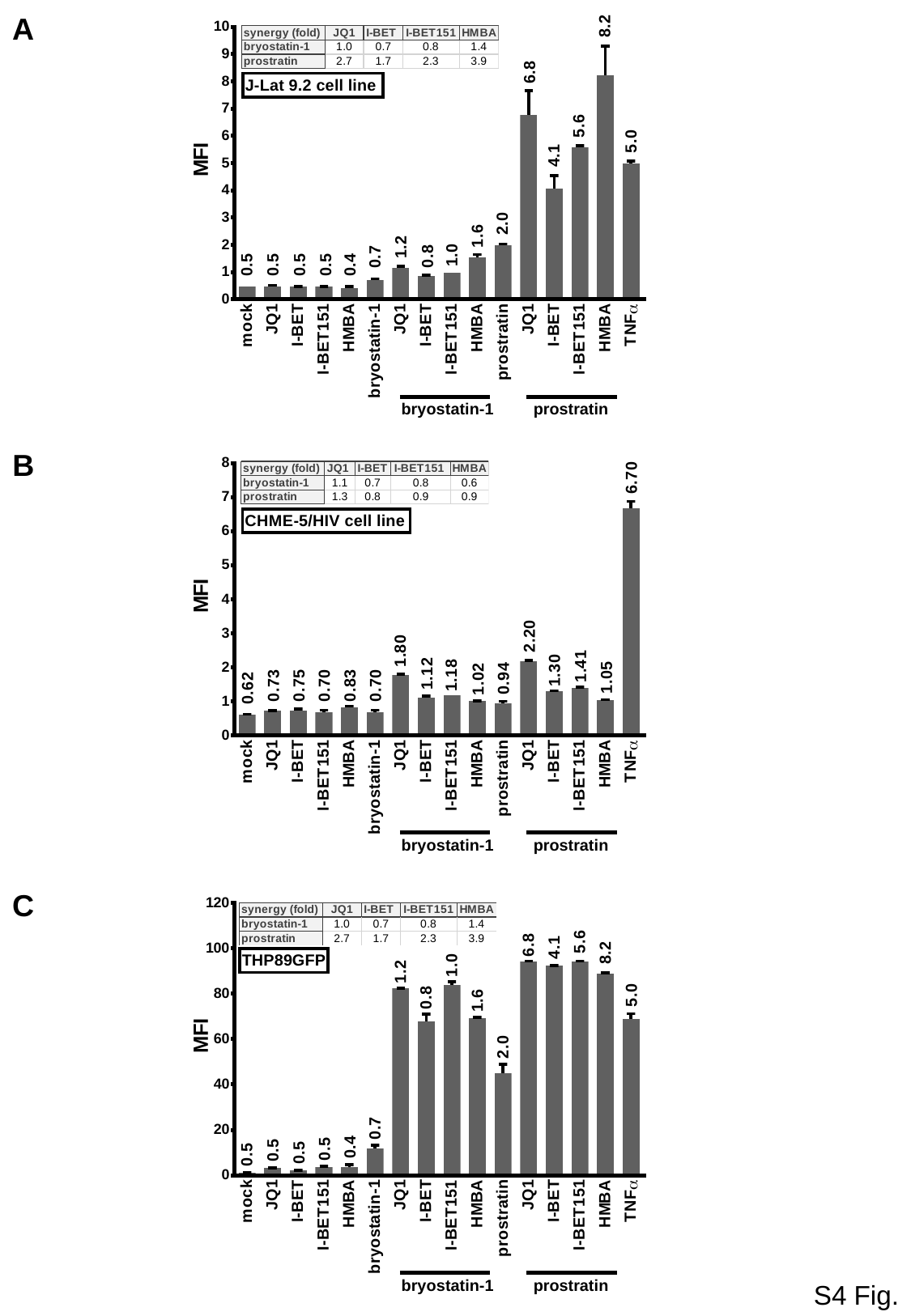

A
B
C
S4 Fig.

Supplement: S4 Fig — The J-Lat 9.2 cell line (panel A), CHME-5/HIV microglial cells (panel B) or THP89GFP monocytic cells (panel C) harbor latent HIV1 provirus containing gfp gene. The cells were mock-treated, treated with JQ1 (0.5μM), I-BET (0.5μM), I-BET151 (0.5μM), HMBA (5mM), bryostatin-1 (10nM) and prostratin (2.5 μM) alone or in combination as indicated. At 24 hours post-treatment, cells were analyzed by flow cytometry and the mean fluorescence intensity (MFI) was analyzed to quantify the amount of GFP produced. Means and standard errors of the means from duplicate samples are indicated. One representative experiment from three is represented. For each combinatory treatment, the fold-synergy was calculated by dividing the effect observed after co-treatments by the sum of the effects after the individual treatments. (PPT) [file ppat.1005063.s004.ppt]

## Slide 1
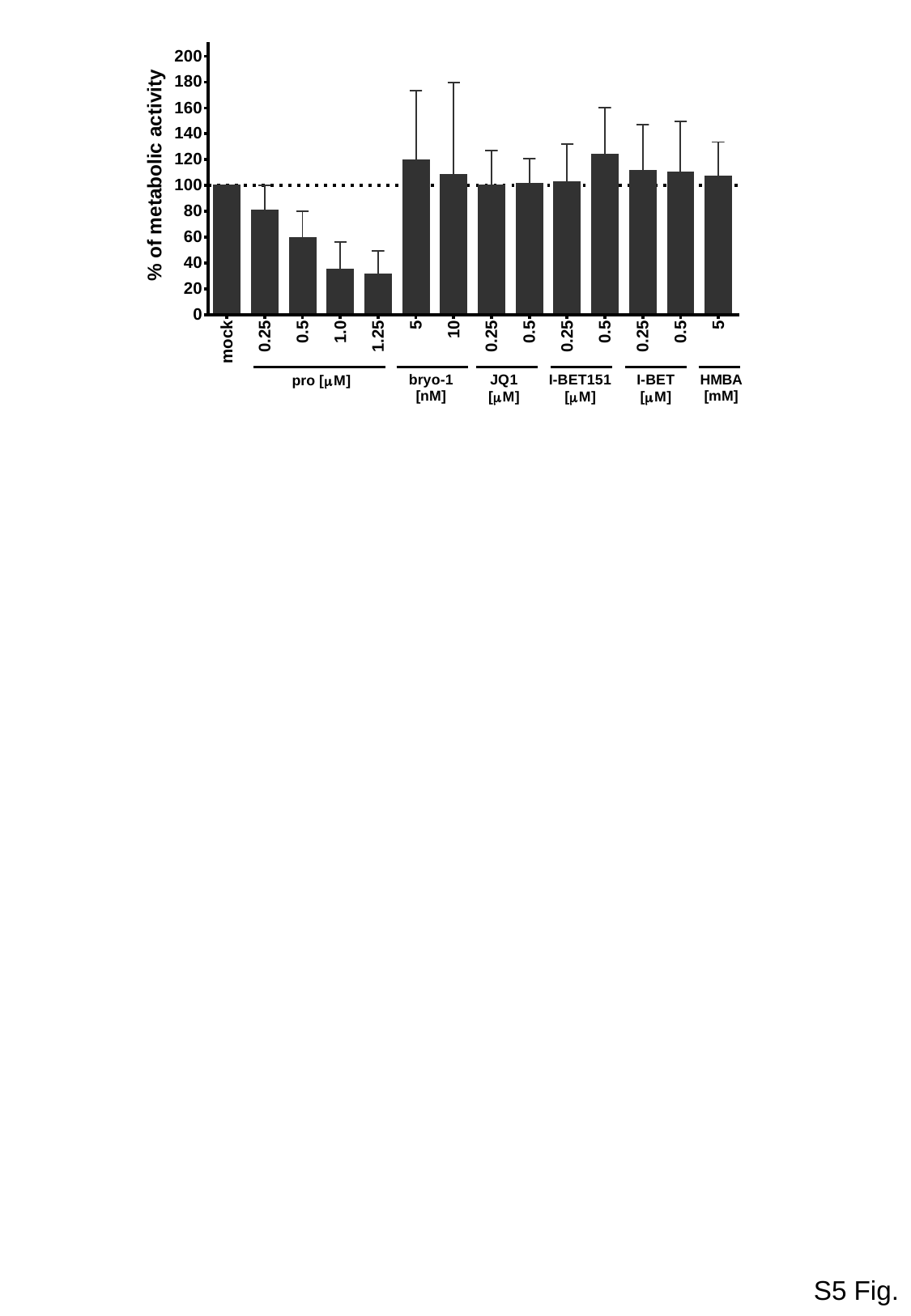

S5 Fig.

Supplement: S5 Fig — WST-1 assay on ex vivo cultures of CD8+-depleted PBMCs isolated from blood of 5uninfected donors were incubated with indicated compounds for 6 days. The result obtained with mock-treated cells was set at a value of 100%. (PPT) [file ppat.1005063.s005.ppt]

## Slide 1
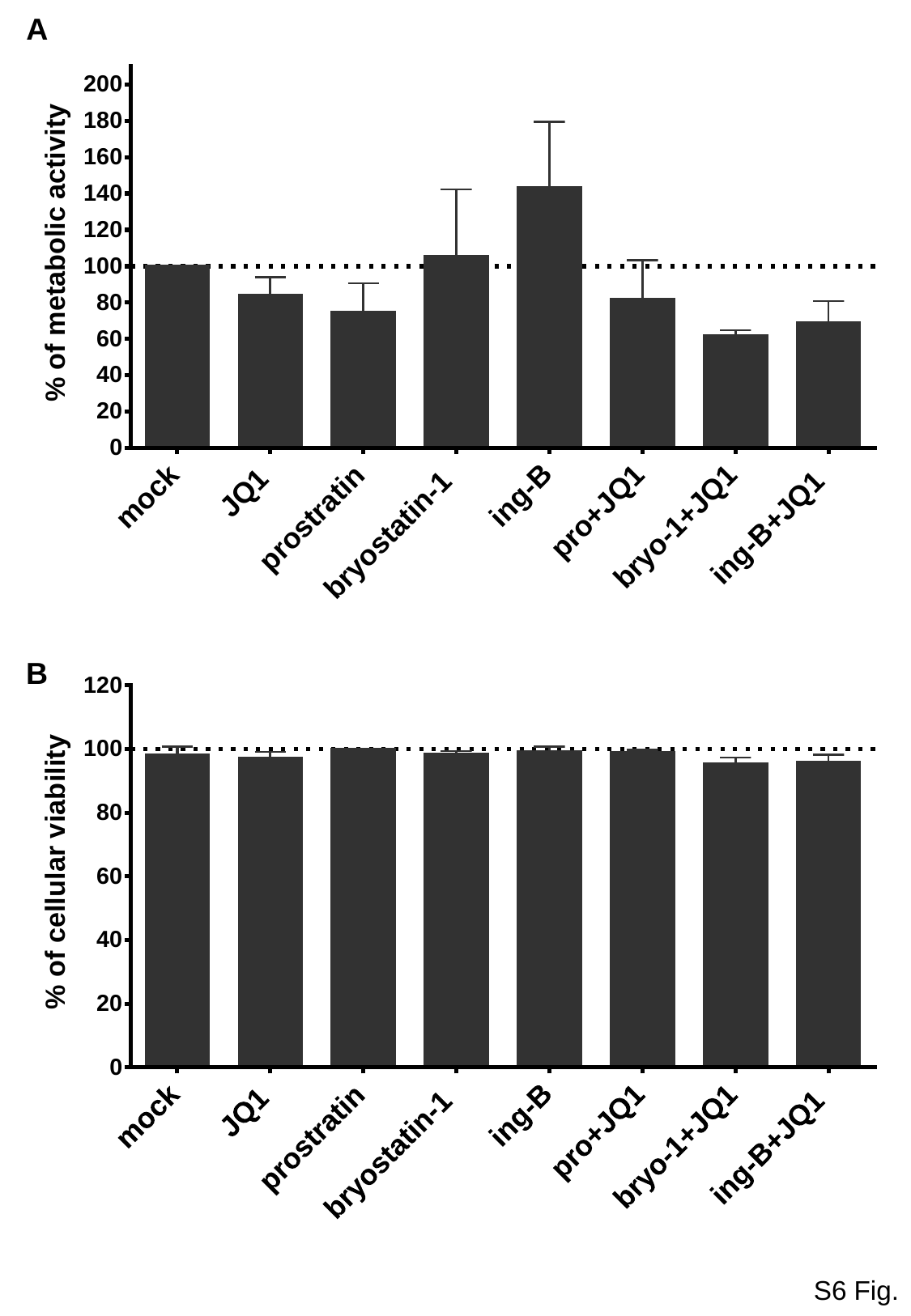

A
B
S6 Fig.

Supplement: S6 Fig — Panel A. WST-1 assay on ex vivo cultures of CD8+-depleted PBMCs isolated from blood of 4 uninfected donors were incubated with indicated compounds for 6 days. The result obtained with mock-treated cells was set at a value of 100%. Panel B. Cell viability. Trypan blue exclusion assay was performed on the same patient cell cultures as described in (A).The result obtained with mock-treated cells was set at a value of 100%. (PPT) [file ppat.1005063.s006.ppt]

## Slide 1
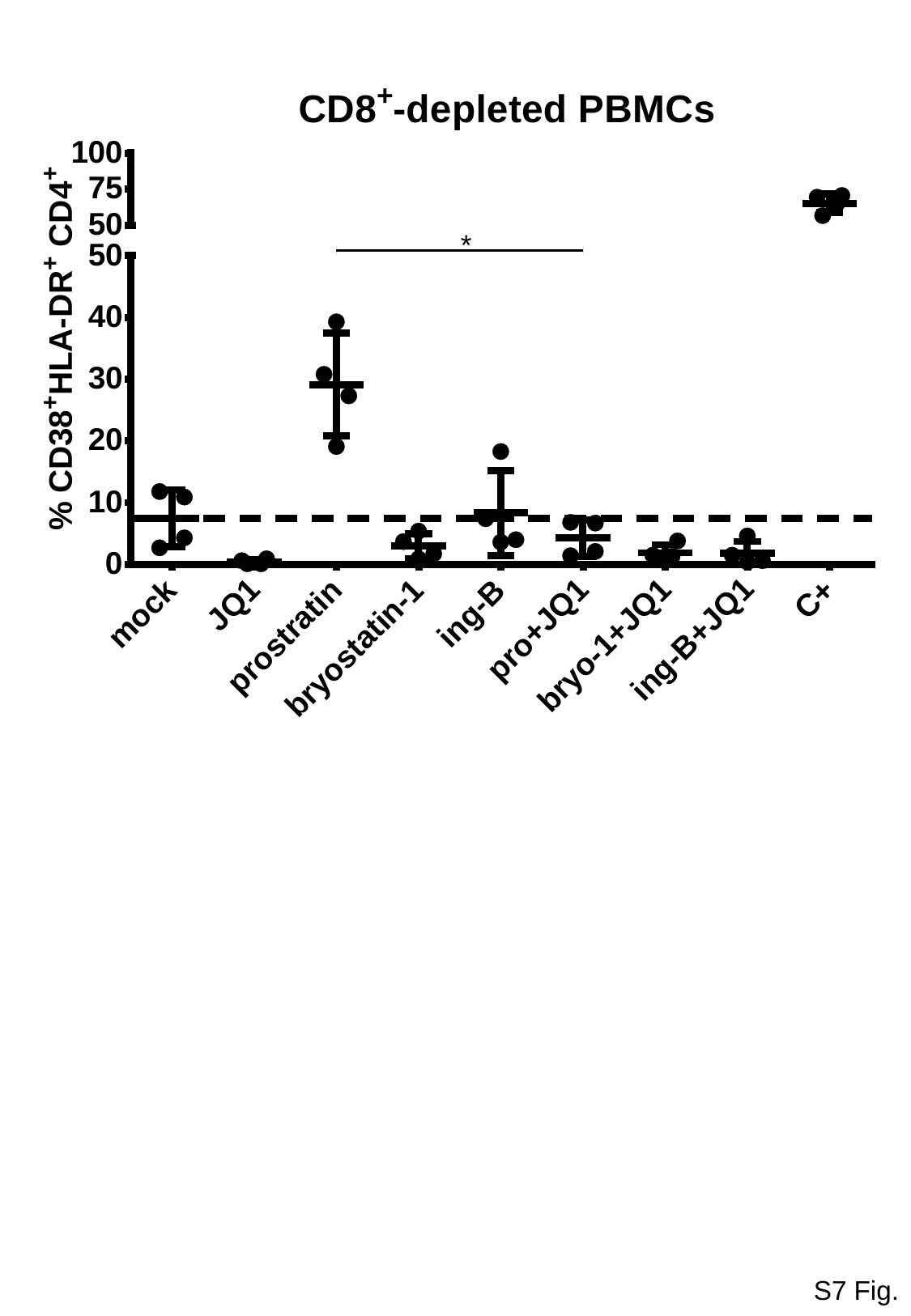

S7 Fig.

Supplement: S7 Fig — CD8+-depleted PBMCs from 4 uninfected donors were mock-treated, treated with anti-CD3+anti-CD28 antibodies (C+), JQ1 (0.25μM), bryostatin-1 (5nM), prostratin (0.5μM) or ingenol B (10nM) alone or in combination for 6 days. Cells were incubated with anti-CD38, anti-HLA-DR, anti-CD4 and anti-CD8 antibodies prior to flow cytometry analysis. The results are presented as percentage of marker expression in the population of CD4+ cells. Dashed line indicates the percentage of expression obtained in mock-treated cells. The means are represented. (PPT) [file ppat.1005063.s007.ppt]

## Slide 1
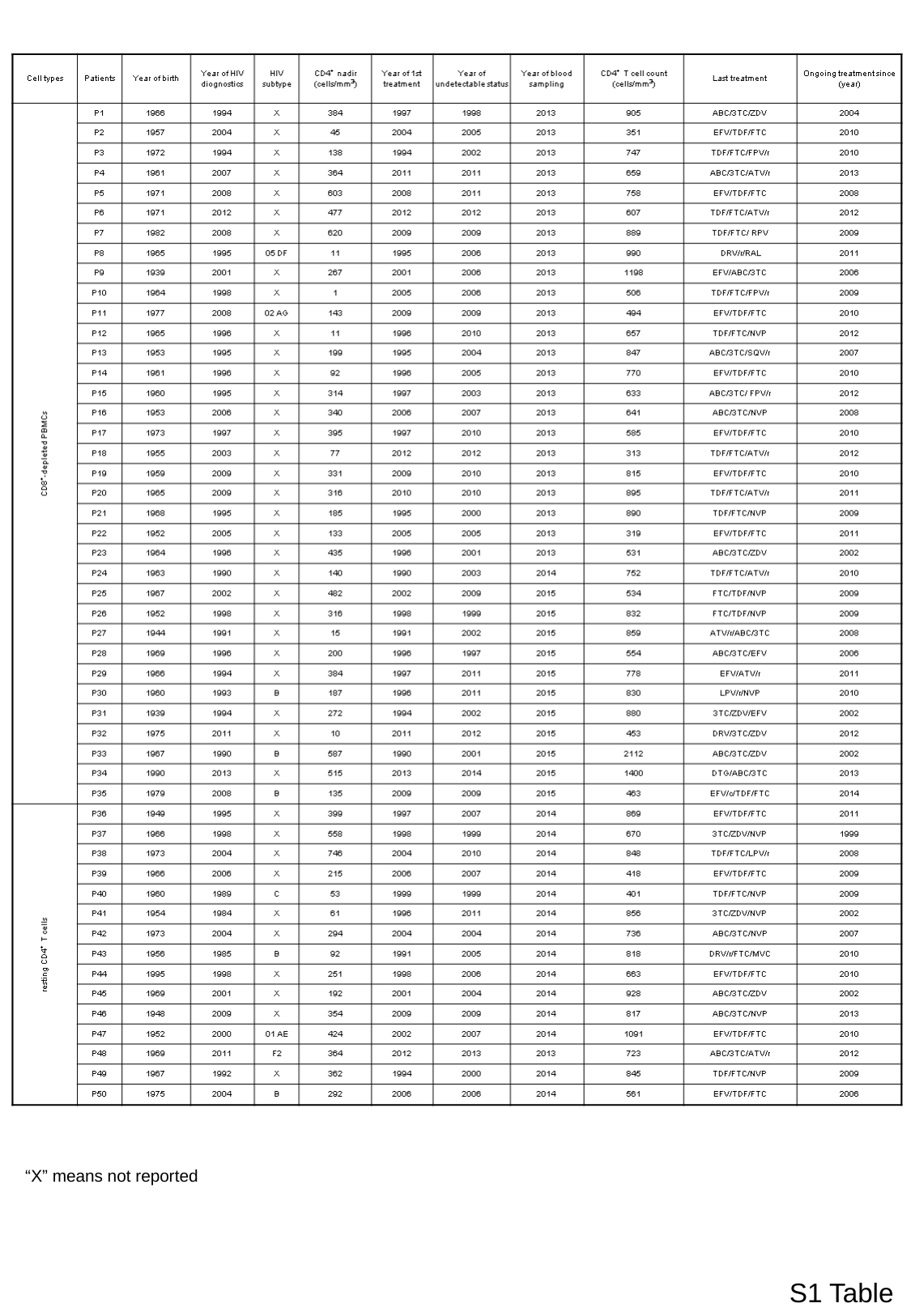

“X” means not reported
S1 Table

Supplement: S1 Table — Characteristics (age, CD4+T cell count, CD4+ nadir, antiviral regimens, duration of therapy, duration with undetectable plasma HIV-1 RNA level, and HIV-1 subtypes) of patients from the St- Pierre Hospital are presented. “X” indicates not reported. (PPT) [file ppat.1005063.s008.ppt]
