# Supplementary material for: An In-Depth Comparison of Latency-Reversing Agent Combinations in Various In Vitro and Ex Vivo HIV-1 Latency Models Identified Bryostatin-1+JQ1 and Ingenol-B+JQ1 to Potently Reactivate Viral Gene Expression
Source: PLoS Pathog. 2015 Jul 30;11(7):e1005063. doi: 10.1371/journal.ppat.1005063 (PMC4520688; doi:10.1371/journal.ppat.1005063)
Supplement: S2 Table — Ex vivo cultures of CD8+-depleted PBMCs from blood of 3 cART-treated HIV+ patient were treated with bryostatin-1+JQ1 for 6 days. Concentrations of viral RNA in culture supernatants were measured and were expressed as HIV-1 RNA copies/ml. Total HIV-1 DNA was expressed as HIV-1 DNA copies/106 CD8+-depleted PBMCs. Viruses collected from ex vivo cell cultures were used to perform de novo infection of Jurkat cells. Six days post-infection, Jurkat culture supernatants were collected and concentrations of viral RNA in culture supernatants were quantified and expressed as HIV-1 RNA copies/ml). (PPT) [file ppat.1005063.s009.ppt]

## Slide 1
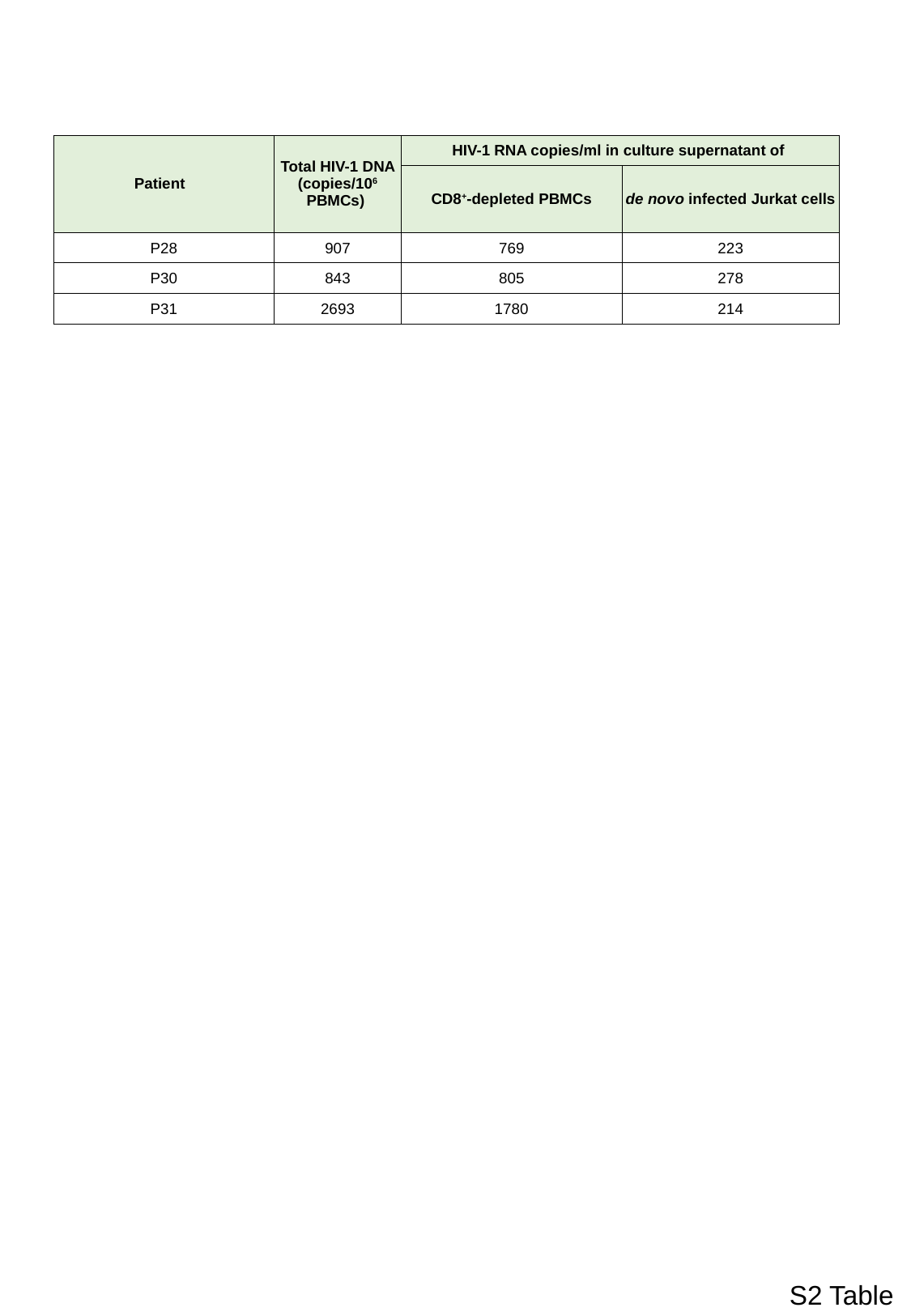

| Patient | Total HIV-1 DNA (copies/106 PBMCs) | HIV-1 RNA copies/ml in culture supernatant of | |
| --- | --- | --- | --- |
| | | CD8+-depleted PBMCs | de novo infected Jurkat cells |
| P28 | 907 | 769 | 223 |
| P30 | 843 | 805 | 278 |
| P31 | 2693 | 1780 | 214 |
S2 Table
